# Who benefits from criminal legal reform? A natural experiment to assess racial disparities in a policy targeting monetary sanctions

**DOI:** 10.1007/s11292-023-09597-3

**Published:** 2024-01-02

**Authors:** Amanda Isabel Mauri, Nancy Nicosia, Beau Kilmer

**Affiliations:** 1 Department of Public Health Policy and Management, New York University, New York, NY USA; 2 RAND Corporation, Santa Monica, CA USA

**Keywords:** Monetary sanctions, Legal financial obligations, Natural experiment, American Indian, Race and ethnicity

## Abstract

**Objective:**

To examine disparities in court fines between American Indian and White convicted persons before and after a South Dakota reform, which trained court personnel to only assess fines that could be reasonably paid by defendants.

**Methods:**

A natural experiment design using criminal records for the universe of convictions for misdemeanor arrests between July 2011–June 2015 (*N* = 34,700) was employed to estimate the association between the reform and the likelihood of a fine using logistic regression.

**Results:**

The reform was associated with reductions in fine assessments in urban (OR, 0.63; CI, 0.39–1.04); rural, no Indian Country (OR, 0.24; CI, 0.18–0.33); and rural, part-Indian Country counties (OR, 0.24; CI, 0.18–0.32). Both American Indian and White persons experienced these reductions, but the reductions were smaller for American Indians in urban counties.

**Conclusions:**

A defendant’s race and features of local court structures may shape judicial behavior in response to monetary sanctioning reforms.

**Supplementary Information:**

The online version contains supplementary material available at 10.1007/s11292-023-09597-3.

## Introduction

American Indian individuals are overrepresented in the criminal legal system relative to their proportion in the general population, yet disparities between American Indian persons and other racial groups are often overlooked in analyses of arrests, convictions, and sanctions. Nationally, American Indian and Alaska Native individuals have the second-highest jail incarceration rate at 420 per 100,000 residents, more than twice as high as the rate among White persons (184 per 100,000 residents) (Zeng & Minton, 2021). Despite calls for research examining sentencing disparities between American Indians and other groups beginning in the 1990s and continuing today (Ulmer & Bradley, 2019), American Indian persons remain a severely understudied population in the criminal legal literature.

Research on monetary legal sanctions reflects the broader literature’s neglect of the experiences of American Indian people, though notable exceptions exist (O’Neill et al., 2022; Stewart et al., 2022). This is particularly important given the substantial evidence demonstrating that the adverse impacts of legal financial obligations are disproportionately borne by low-income people of color, including American Indian persons, due to disparities in income and criminal legal outcomes between racial and ethnic minorities and non-Hispanic White persons (Bhutta et al., 2020; Carson, 2018, 2020; Semega et al., 2021). Failing to pay legal financial obligations may result in parole or probation revocations, potentially triggering incarceration; driver’s licensure suspensions, impacting one’s ability to commute for employment, school, or health care; and poor credit scores, compromising access to good housing, employment, and loans, among other harms (Harris, 2016; Harris et al., 2011; Martin et al., 2018).

In this paper, we use a natural experiment and unique criminal legal data from South Dakota to estimate racial disparities in fines—one type of monetary sanction—before and after a reform to the state’s monetary sanctioning system. South Dakota offers an excellent opportunity to examine racial disparities in financial penalties between American Indian and White persons. First, American Indian persons make up an important share of the South Dakota population (11.1% American Indian or Alaskan Native alone or in combination with another race and ethnicity; 8.8% American Indian or Alaskan Native alone) (United States Census Bureau, 2020). Second, American Indian persons are severely overrepresented in the South Dakota criminal legal system, as they are nationally; in 2016, the South Dakota jail incarceration rate per 100,000 was 162 for White people, 1052 for Black people, and 1503 for American Indian people (Incarceration Trends in South Dakota, 2019).

Finally, South Dakota’s Public Safety Improvement Act of 2013 (hereafter, SB70) created a natural experiment to assess changes in financial penalties due to a reform to the state’s monetary sanctioning practices. In addition to reforms in sentence length for felony offenses and financial sanctions for all offenses, SB70 required that the South Dakota Unified Judicial System establish a financial accountability system to monitor and collect financial obligations ordered by the court (Improve Public Safety, 2013). This system included an “ability-to-pay” policy that trained judges and other court personnel to only assess fines and other legal fees at amounts realistically payable by the defendant. Thus, the South Dakota reform offers a rare natural experiment to assess whether a policy intended to universally reduce monetary sanctions produced heterogeneous effects by race.

## Background

Researchers have long studied localized inequities in sanctions following the implementation of sentencing reforms (Bernard & Engel, 2001; Dixon, 1995; Myers & Talarico, 2012). While the overarching objective of these policies is to limit autonomy by imposing sentencing constraints and norms, most leave room for judicial discretion. Put another way, these policies create frameworks by which judges adjust sentences to reflect the circumstances of the defendant and the court, creating variation in sentencing practices across communities (Eisenstein et al., 1988; Ulmer, 1997).

But sentencing policies offer varying degrees of discretion. Some policies constrain autonomy by imposing narrow boundaries on available penalties, whereas others broaden judicial discretion. “Ability-to-pay” policies require judges to adjust fines according to the socioeconomic status of the defendant, increasing judicial autonomy by providing the judge latitude in determining which minimum standards, like income, public assistance, housing security, transportation access, and financial penalties from other courts to consider (Fernandes et al., 2019; Shannon et al., 2020); what weights to put on each measure; and how these metrics impact the decision to assess a fine and fine size. Our objective is to examine if this expansion of judicial discretion curtails, maintains, or exacerbates racial disparities in fine assessment between American Indian and White convicted persons.

On one hand, the ability-to-pay consideration might disproportionately benefit American Indian defendants. Since American Indian persons have substantially lower household incomes, on average, than White persons, American Indian convicted persons should more greatly benefit from this reform relative to White individuals. On the other hand, researchers have shown that some sentencing reforms that increase judicial discretion do not impact disparities in sentencing outcomes (Ulmer et al. 2011a, b). For instance, court actors may have already been considering ability-to-pay when assessing fines, implying that the policy simply institutionalized an existing practice.

In addition, the reform does not impact any of the extralegal factors in sentencing decisions. Several frameworks illustrate how biases associated with the defendant’s characteristics—features that are theoretically legally irrelevant—affect treatment in criminal legal processes. One such model is the focal concerns perspective (Steffensmeier et al., 1998; Ulmer, 2019), which argues that court actors incorporate judgments about an offender’s blameworthiness, practical constraints, and dangerousness into punishment decisions. When making these determinations, court actors rely both on legally relevant information and stereotypes about groups as perceptual short-hands to guide their decision-making (Skolnick, 1966). Importantly, the ability-to-pay policy examined here does not impact these extralegal considerations.

As Ulmer and Bradley (2019) note, stereotypes about American Indian persons may impact their treatment in state courts in harmful or beneficial ways. American Indian individuals are the victims of negative stereotypes related to the high prevalence of substance use in their communities and, as a result, the likelihood of perpetuating crime as a result of substance use (Alvarez & Bachman, 1996; Franklin, 2013). These stereotypes may lead court actors to judge that American Indian persons are more blameworthy or dangerous than White individuals, thus producing more severe punishments for American Indian defendants. In another scenario, American Indian persons may receive lesser sanctions because of their disadvantaged circumstances. Indeed, court actors might perceive American Indian convicted persons as less blameworthy for their offenses because of the structural disadvantages they face in education, employment, healthcare, housing, and politics, thus resulting in more lenient judicial treatment (Jeffries & Bond, 2012).

Importantly, these judgments about the blameworthiness, practical constraints, and dangerousness of American Indian persons likely depend on place. Many have argued that comparing American Indian punishment outcomes to other groups exclusively through a racial/ethnic lens is incomplete. Rather, sentencing disparities between American Indian and other groups may vary by, for example, whether the community’s roots intertwine with the historic economic and social colonial suppression of American Indian communities (Steinman, 2012; Ulmer & Bradley, 2019). Stewart et al. (2022) describe how trends in more individualized debt collection methods—like fines, restitution, and court fees—may follow local histories related to the collective imposition of debt on American Indian communities. Empirically, the authors find support for this claim, observing that legal financial obligations are more severe in communities made up of or proximal to Indian Country in Minnesota than in communities with no Indian Country. At the same time, we may also envision a scenario where punishments are less severe in communities with Indian Country. Court actors in communities neighboring Indian Country may be particularly attuned to the oppression of American Indian people. Consequently, judges might elect to punish American Indian defendants less severely because they consider that their historic and structural disadvantage makes them less blameworthy than other groups.

Taken together, SB70’s ability-to-pay policy expanded the judicial discretion in sentencing decisions by requiring judges to assess the defendants’ socioeconomic characteristics when deciding whether to assess a fine and determining a fine amount. As noted, since American Indian individuals are more likely to be of lower income than White persons, this policy may greatly benefit American Indian defendants. At the same time, the policy may maintain existing disparities in sentencing practices because it does not change the extralegal considerations factored into judicial decisions. This study examines whether SB70’s ability-to-pay policy maintained, exacerbated, or reversed differences in the likelihood of receiving a fine between American Indian and White persons.

## Methods

Criminal record data from the South Dakota Attorney General’s Office contain information on all arrestees ages 18 and older. The record tracks each arrest from the moment of arrest through disposition and, when applicable, sentencing. Our unit of analysis is arrests made between July 1, 2011, and June 30, 2015—two years before and two years after SB70’s July 1, 2013, effective date (eFigure 1 for the analysis sample flowchart). Our analysis period ended in mid-2015 to avoid potential confounding with the creation of an Obligation Recovery Center (ORC), which involved contracting out debt collection to a private agency (Obligation Recovery Center, 2015).

Our sample of arrests was further restricted based on criminal legal and socio-demographic characteristics. To explore racial disparities in fine assessment, we restricted our cohort to those individuals arrested for a single misdemeanor charge that resulted in a conviction. We excluded arrests with a felony charge for two reasons: (1) misdemeanors were substantially more likely to be sanctioned with a fine than arrests with a felony conviction before (72.47% vs 29.75%) and after the reform (47.73% vs 17.17%), and (2) other SB70 elements targeted felonies, so changes in penalties for felony convictions may be associated with the financial accountability system and/or other provisions. By limiting our analysis to arrests with a misdemeanor conviction only, which were largely unaffected by SB70’s other provisions, our analysis provides a cleaner estimate of the association between the reform of the financial accountability system and fine assessment. We further excluded misdemeanor convictions with a penalty other than jail only or jail and fine penalty. Convictions with other penalty types (e.g., prison and fine, prison only, fine only) made up less than 2% of all misdemeanor convictions. We also excluded convictions with missing penalty information (7.7%).

Given South Dakota’s demographic composition, we limited the cohort to convictions where the individual’s race was recorded as American Indian or White. This criterion excluded less than 10% of remaining convictions. Our analysis also excluded arrests in the six counties comprised entirely of Indian Country, based on the federal definition, which includes federal Indian reservations and trust land allotments (18 USC § 1151, 1949; 40 CFR § 171.3, 2017) (eFigure 2 for map of Indian Country in South Dakota). This exclusion affected less than 1% of misdemeanor convictions and aligns with our expectation given the “jurisdictional maze” that characterizes legal jurisdiction in Indian Country (Deloria & Lytle, 1983; Ulmer & Bradley, 2018, 2019). Jurisdiction for crimes occurring in South Dakota’s Indian Country lies with either tribal or federal courts when the defendant is American Indian (Leonhard, 2011). While crimes involving a non-American Indian defendant occurring in Indian Country may be prosecuted in federal or state court depending on the crime (Droske, 2007), our data demonstrate that the latter is rare.

All analyses are stratified by the intersection of rurality and Indian Country, resulting in three samples based on the arrest county: (1) urban; (2) rural, no Indian Country; and (3) rural, part-Indian Country. We stratify based on the intersection of rurality with Indian Country because of research demonstrating that rural criminal systems are associated with more severe legal punishments, including monetary sanctions (O’Neill et al., 2022; Olson & Ramker, 2001; Ruback & Clark, 2011). All models are adjusted for individual-level demographic and criminal legal characteristics including age, sex, number of previous arrests, charge type (e.g., drug, property, violent, DUI charge), and jail sentence length. All individual-level covariates are constructed from criminal records. We also include the county’s monthly unemployment rate from the Bureau of Labor Statistics as a county-level covariate (United States Bureau of Labor Statistics, n.d).

One important issue is whether reductions in the prevalence of fines were accompanied by changes in fine amounts. For example, judges may have reduced the size of fines assessed in addition to or rather than reducing their prevalence. On the other hand, judges may have reduced the number of fines, but increased the size of fines, resulting in minimal change in the total revenue earned by the county and state. If such a positive association exists, it may be more substantial in rural counties, which were more likely to issue fines than urban counties pre-reform. To address the concern that judges may have adjusted fine size after the reform, we also provide a supplemental analysis at the same arrest level that examines the association between the fine amount and the reform.

The project was approved by the Institutional Review Board at the RAND Corporation. All analyses were completed with STATA version 16.0 (StataCorp, College Station, TX), and all regression models cluster standard errors by county month–year.

## Results

Table 1 demonstrates that the demographic and criminal legal characteristics of the 34,700 convictions in our cohort were similar in the period before and after the reform across county types (see eTables 1A–1C for demographic and criminal legal characteristics, stratified by county type). The number of convictions was similar in the 2-year period pre-reform (*N* = 17,721) and post-reform (*N* = 16,979). American Indian persons comprised 31.0% of the sample arrests between July 1, 2011 and June 30, 2013, and 34.2% between July 1, 2013 and June 30, 2015. More convictions were associated with arrests in urban and rural, no Indian Country counties than in rural, part-Indian Country counties both before and after the reform.

**Table 1:** Demographic and criminal legal characteristics of misdemeanor convictions in South Dakota

	July 1, 2011–June 30, 2013				July 1, 2013–June 30, 2015			
	American Indian		White		American Indian		White	
	*N*	%	*N*	%	*N*	%	*N*	%
Penalty type								
Jail only	2321	42.21%	2440	19.96%	3367	57.98%	5346	47.85%
Jail and fine	3178	57.79%	9782	80.04%	2440	42.02%	5826	52.15%
Sex								
Male	3543	64.43%	8908	72.88%	3794	65.33%	8083	72.35%
Female	1956	35.57%	3314	27.12%	2013	34.67%	3089	27.65%
Age								
18–24	1779	32.35%	4074	33.33%	1712	29.48%	3428	30.68%
25–34	1717	31.22%	3502	28.65%	1854	31.93%	3173	28.40%
35–44	1039	18.89%	2082	17.03%	1109	19.10%	1997	17.88%
45 +	964	17.53%	2564	20.98%	1132	19.49%	2574	23.04%
Prior arrest								
0	980	17.82%	4620	37.80%	843	14.52%	4029	36.06%
1	667	12.13%	2268	18.56%	645	11.11%	2036	18.22%
2	540	9.82%	1383	11.32%	559	9.63%	1241	11.11%
3 +	3312	60.23%	3951	32.33%	3760	64.75%	3866	34.60%
Charge type								
Drug	316	5.75%	718	5.87%	353	6.08%	666	5.96%
DUI	1243	22.60%	5796	47.42%	1120	19.29%	5051	45.21%
Property	1197	21.77%	1849	15.13%	1356	23.35%	1925	17.23%
Violent	800	14.55%	1763	14.42%	961	16.55%	1774	15.88%
Other	1943	35.33%	2096	17.15%	2017	34.73%	1756	15.72%
Jail sentence length (days)								
1–30	3509	63.81%	7661	62.68%	3783	65.15%	7354	65.83%
31–60	728	13.24%	1820	14.89%	726	12.50%	1411	12.63%
61–90	600	10.91%	1300	10.64%	606	10.44%	1053	9.43%
91 +	662	12.04%	1441	11.79%	692	11.92%	1354	12.12%

**Fig. 1:**
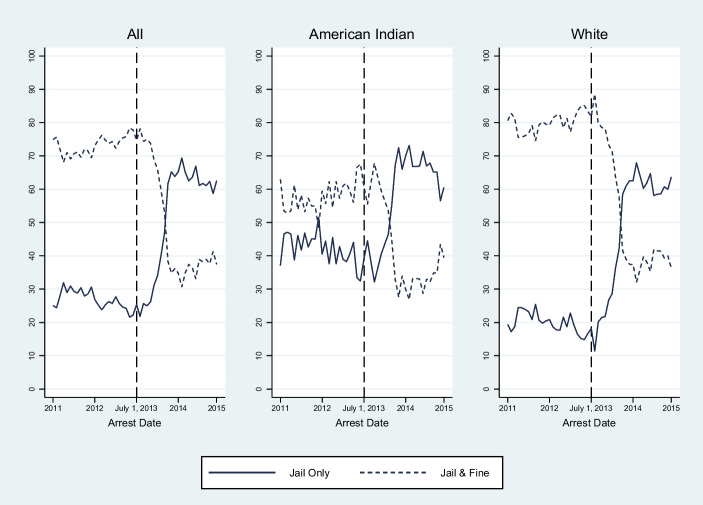
Percent of misdemeanor convictions assessed a fine in South Dakota. The figures depict the percent of misdemeanor convictions that assessed a fine 2 years before and after the reform’s July 1, 2013, effective date

The proportion of these convictions that received a fine decreased substantially after the reform became effective on July 1, 2013 (Fig. 1) (eFigures 3A–3C for trends in fine assessment, stratified by county type). Before the reform, 73.1% of convictions were assessed a fine. That share declined to 61.0% during the first year after the reform and subsequently declined to only 36.5% during the second year after the reform. The pattern was similar across all county types, but the percentage of convictions that received a fine was greater in rural counties than in urban counties pre-reform (urban, 64.7%; rural, no Indian Country, 83.0%; rural, part-Indian Country, 81.4%) and remained so post-reform (urban, 42.2%; rural, no Indian Country, 55.0%; rural, part Indian Country, 58.0%).

The results from the logistic regression depicting the association between the reform and fine assessment are presented as odds ratios with 95% CI in Table 2 (eTables 2A–2B for the full results and eTable 2C for marginal effects). The reform was associated with lower odds of receiving a fine in all county types post-reform (urban—OR, 0.63; CI, 0.39–1.04; rural, part Indian Country—OR, 0.24; CI, 0.18–0.33; rural, no Indian Country—OR, 0.24; CI, 0.18–0.32) (Table 2 Panel A). When we disaggregate the post-reform period into 6-month intervals, the lower odds manifested after the initial 6-month period in all county types (Table 2 Panel B).

**Table 2:** Association between South Dakota reform and fine assessment

	Urban	Rural, no Indian country	Rural, part-Indian country
	OR (95% CI)	OR (95% CI)	OR (95% CI)
Panel A: binary reform indicator			
Reform (ref. pre-reform)	0.63 (0.39–1.04)	0.24** (0.18–0.33)	0.24** (0.18–0.32)
Panel B: 6-month interval reform indicator			
Reform (ref. pre-reform)			
6 months post	0.79 (0.53–1.17)	1.19 (0.86–1.65)	0.76 (0.52–1.11)
12 months post	0.31** (0.20–0.47)	0.14** (0.09–0.20)	0.17** (0.11–0.26)
18 months post	0.12** (0.07–0.20)	0.08** (0.06–0.12)	0.13** (0.09–0.19)
24 months post	0.18** (0.10–0.31)	0.09** (0.06–0.13)	0.12** (0.08–0.19)
Observations	18,392	10,225	6061

Results were produced using logistic regression models with the following control variables: gender, age, prior arrests, jail sentence length, charge type, and county unemployment, as well as county and month-of-year fixed effects. All standard errors are clustered by county and month–year. The periods for the binary indicators are pre-reform (July 1, 2011–June 30, 2013) and post-reform (July 1, 2013–June 30, 2015). The periods for the 6-month interval indicator refer to the following dates: pre-reform, July 1, 2011–June 30, 2013; 6 months post, July 1, 2013–December 31, 2013; 12 months post, January 1, 2014–June 30, 2014; 18 months post, July 1, 2014–December 31, 2014; 24 months post, January 1, 2015–June 30, 2015

^**^p < 0.01; *p < 0.05

Table 3 demonstrates heterogeneity in the reduction in odds by race across county types (eTables 3A–3B for the full results and eTable 3C for marginal effects). The main effects imply lower odds post-SB70 in urban (OR, 0.45; CI, 0.27–0.73); rural, no Indian County (OR, 0.22; CI, 0.16–0.29); and rural, part-Indian Country (OR, 0.22; CI, 0.15–0.31) counties (Table 3 Panel A). The interaction terms between the reform and race suggest the reductions in odds were larger in magnitude for American Indian than White defendants in rural, no Indian Country (albeit imprecise) (OR, 0.86; CI, 0.73–1.02) and rural, part-Indian Country counties (OR, 0.71; CI, 0.57–0.88), but smaller in magnitude for American Indians than White persons in urban counties (OR, 1.22; CI, 1.02–1.44). The results remain robust to the inclusion of a main effect for race (eTables 4A–4B) and other race/ethnic groups in the expanded sample (eTables 4C–4F), in a three-way interaction model in lieu of stratification (eTables 4G), to a multi-level model framework (eTable 4H), and multiple imputation for the observations dropped due to missing penalty information (eTables 4I-4L).

**Table 3:** Association between South Dakota reform, race, and fine assessment

	Urban	Rural, no Indian country	Rural, part-Indian country
	OR (95% CI)	OR (95% CI)	OR (95% CI)
Panel A: binary reform indicator			
Reform (ref. pre-reform)	0.45** (0.27–0.73)	0.22** (0.16–0.29)	0.22** (0.15–0.31)
Reform*AI	1.22* (1.02–1.44)	0.86 (0.73–1.02)	0.71** (0.57–0.88)
Panel B: 6-month interval reform indicator			
Reform (pre-reform)			
6-months post	0.73 (0.48–1.11)	1.23 (0.88–1.73)	0.78 (0.49–1.25)
12-months post	0.21** (0.13–0.33)	0.12** (0.08–0.17)	0.15** (0.09–0.23)
18-months post	0.08** (0.04–0.13)	0.07** (0.05–0.10)	0.12** (0.07–0.18)
24-months post	0.10** (0.06–0.17)	0.07** (0.04–0.11)	0.11** (0.07–0.19)
Reform*AI			
6 months post	0.60* (0.40–0.91)	0.44** (0.29–0.68)	0.55* (0.35–0.87)
12 months post	1.18 (0.85–1.64)	0.86 (0.61–1.19)	0.77 (0.52–1.15)
18 months post	1.65** (1.19–2.29)	0.88 (0.58–1.32)	0.72 (0.49–1.06)
24 months post	1.97** (1.41–2.74)	1.13 (0.80–1.59)	0.68* (0.46–1.00)
Observations	18,392	10,225	6061

AI = 1 if the convicted person’s race was recorded as American Indian and 0 if the recorded race was White. Results were produced using logistic regression models with the following control variables: gender, age, prior arrests, jail sentence length, charge type, and county unemployment, as well as county and month-fixed effects. The periods for the binary indicator are pre-reform (July 1, 2011–June 30, 2013) and post-reform (July 1, 2013–June 30, 2015). The periods for the 6-month interval indicator refer to the following dates: before the reform, July 1, 2011–June 30, 2013; 6 months post, July 1, 2013–December 31, 2013; 12 months post, January 1, 2014–June 30, 2014; 18 months post, July 1, 2014–December 31, 2014; 24 months post, January 1, 2015–June 30, 2015.

*AI* American Indian

^**^p < 0.01; *p < 0.05

An interesting aspect of these results is their evolution during the first 2 years. During the first 6 months post-reform, the interaction effects suggest that SB70 was associated with greater reductions in fines for American Indian persons (relative to Whites) for all county types (Table 3 Panel B). But by 18 months post-reform, the interaction effects indicate lesser reductions for American Indian persons in urban counties (OR, 1.65; CI, 1.19–2.28), whereas the interaction effects were largely insignificant in the second year in both rural, no Indian Country (OR, 0.88; CI, 0.58–1.32) and rural, part Indian Country (OR, 0.72; CI, 0.49–1.06) county types.

In the full model specifications (eTables 2A–2B and 3A–3B), several covariates are associated with the likelihood of fine assessment. Convictions for a DUI charge increased the odds of receiving a fine in all county types. In addition, individuals aged 45 or older consistently have lower odds than 18- to 24-year-olds in urban counties, but not in rural counties.

The goal of South Dakota’s reform was to create a more equitable system for monetary sanctioning that considered the burden of a financial penalty. While we do not have access to individuals’ income or employment data, we attempt to examine the relationship between fine assessment and the economic environment by controlling for the county-level monthly unemployment rate. If judges were sensitive to the jurisdiction’s socioeconomic environment, we would observe an odds ratio coefficient on the unemployment rate that is less than one; as the county unemployment rate increased, judges would be less likely to assess a fine. The coefficient and significance of the unemployment rate variable vary by county type (eTables 2A–2B, 3A–3B). When we disaggregate the post-reform period into 6-month intervals, we observe an odds ratio of less than one in rural counties, but an insignificant coefficient in urban counties. In our supplementary analyses, we also found no evidence that the reform was associated with an increase in fine size (eTable 5), suggesting that total revenues from fines likely decreased post-reform.

## Discussion

To our knowledge, this is the first study to evaluate a natural experiment of an “ability-to-pay” reform on racial disparities in monetary sanctions. While the prevalence of convictions assessed a fine declined after implementing the financial accountability system (by 50% in the second year), those changes were not equally distributed across counties and racial groups. Before the reform, American Indian persons were less likely to receive a fine than White individuals across all county types. One year after the reform’s implementation, the likelihood of receiving a fine decreased more for White than American Indian persons in urban counties, so much so that American Indian convicted individuals became more likely to receive a fine than White convicted persons. In contrast, American Indian and White persons experienced similar reductions in fine assessment in rural counties, meaning that American Indian convicted individuals remained less likely to receive a fine than White counterparts in these counties.

An important limitation of this study is that our data do not facilitate an evaluation of the factors generating this divergent response to the reform. Put another way, while we observe different responses, our data and methodological approach limit our ability to examine the production of these disparities. However, insights from existing literature inform what may drive the observed disparities by place and race.

As a reminder, American Indian convicted persons were less likely to receive a fine than White persons both before and after the reform in rural counties. While this finding contrasts with previous research demonstrating that American Indian defendants bear more severe sanctions in rural counties (Stewart et al., 2022), it aligns with scholarship suggesting that judges in rural communities may have already been incorporating the lower socioeconomic status of American Indian persons into their decision making before the reform. Court actors in rural legal systems are more attuned to a convicted person’s personal circumstances because of heightened familiarity among residents (Kirk et al., 2022; Statz, 2021). Our findings suggest that a judge in a rural community may not require a formal ability-to-pay policy to consider a person’s socioeconomic status. In our case, a judge may have already been incorporating the lower ability-to-pay of American Indian persons relative to White persons into fine assessment given the social intimacy characteristic of rural communities.

In addition, our data does not offer an answer to why American Indian convicted persons became more likely to be assessed a fine than White persons in urban communities after the reform. However, the higher likelihood of a fine suggests that negative stereotypes about American Indian persons may play a role. American Indian individuals may face prejudice that they are more dangerous and/or blameworthy for their crimes than their White peers (Alvarez & Bachman, 1996; Franklin, 2013; Jeffries & Bond, 2012). Ability-to-pay policies may activate these biases by requiring judges to adjust fees based on the defendant’s characteristics. Put another way, by requiring judges to consider the socioeconomic circumstances of the defendant, judges may be more likely to incorporate prejudices informed by these circumstances into sentencing decisions. Consequently, judges with these negative stereotypes may be more likely to use ability-to-pay considerations to eliminate fees for White persons than American Indian persons.

Further, our research does not inform what aspects of the financial accountability system resulted in reduced fines. Past research suggests that, in addition to training, administrative guidance, and regulatory oversight, a policy’s success in creating uniformity in sentencing practices also depends on perceptions of its legitimacy. If court actors view a policy as based on best practices or simplifying complex processes, they may be more likely to conform to its prescriptions (Ulmer, 2019). Qualitative research, such as Smith et al. (2022) study interviewing and observing court personnel after reforms to monetary sanctioning laws, is better equipped to unpack what aspects of monetary sanctioning policies, including policy perceptions, affect how judges decide when to issue and how to structure monetary sanctions.

There are a few notable limitations of this study. First, we do not have access to some individual-level variables (e.g., income) that might inform decisions about individuals’ ability to pay. As with most criminal legal data, we only have access to basic demographics, such as race and sex, and the socioeconomic characteristics of the county. We are thus unable to probe more deeply into the extent to which various factors may inform these findings. Our data also relate to a single state and so may not be generalizable to other states that differ in their state and county governance or other structures. Further, due to potential confounding by other policy changes in the state in mid-2015, we are unable to track effects beyond the 2-year period.

Additional expansions of our work include examining changes in other legal financial obligations, like retribution or court-ordered fees, before and after the reform. If other legal sanctions increased, the financial accountability system may not have reduced financial burdens but shifted it to another collection mechanism. Further, the financial accountability system creates an opportunity to compare outcomes of similar populations with divergent likelihoods of fine assessment. The population associated with misdemeanor convictions 2 years before and after July 1, 2013, have similar demographic and criminal legal profiles but drastically different probabilities of receiving a fine. Thus, the financial accountability system creates a rare natural experiment suited to address the lack of robust quantitative research examining the effect of monetary sanctions on outcomes ranging from recidivism and political participation to educational attainment, housing insecurity, and health status.

## Conclusion

Jurisdictions around the country are increasingly implementing policies that explicitly aim to reduce financial penalties (Harvard Law School, 2023). Our research demonstrates that those policies may influence existing criminal legal disparities. To equitably achieve the intent of monetary sanctioning reforms, policymakers, implementers, and researchers should monitor how legal and extralegal considerations - such as the defendant’s race and ethnicity and local court structures - produce varying experiences of criminal legal policies.

## Supplementary Information

Below is the link to the electronic supplementary material.

Supplementary file1 (DOCX 727 KB)
