# Supplementary material for: Who benefits from criminal legal reform? A natural experiment to assess racial disparities in a policy targeting monetary sanctions
Source: J Exp Criminol. 2024 Jan 2;21(2):451–64. doi: 10.1007/s11292-023-09597-3 (PMC12222349; doi:10.1007/s11292-023-09597-3)
Supplement: Supplementary file 1 — Supplementary file1 (DOCX 727 KB) [file 11292_2023_9597_MOESM1_ESM.pdf]

Supplemental Materials

Who Benefits from Criminal Legal Reform? A Natural Experiment to Assess Racial Disparities in a Policy Targeting Monetary Sanctions

Amanda I. Mauri, Nancy Nicosia, Beau Kilmer

eFigure 1: Sample Eligibility

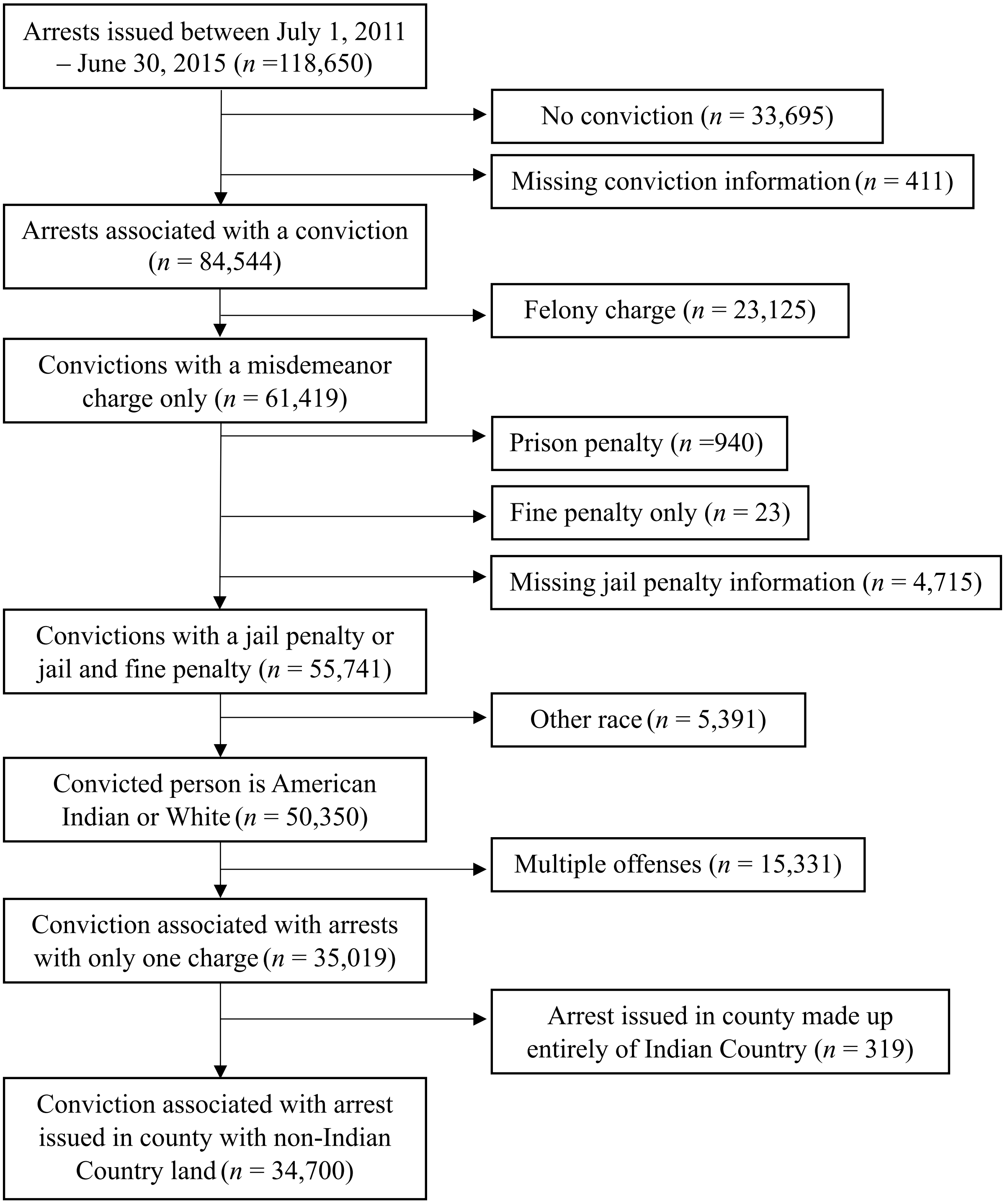

eFigure 2: Map of Indian Country in South Dakota

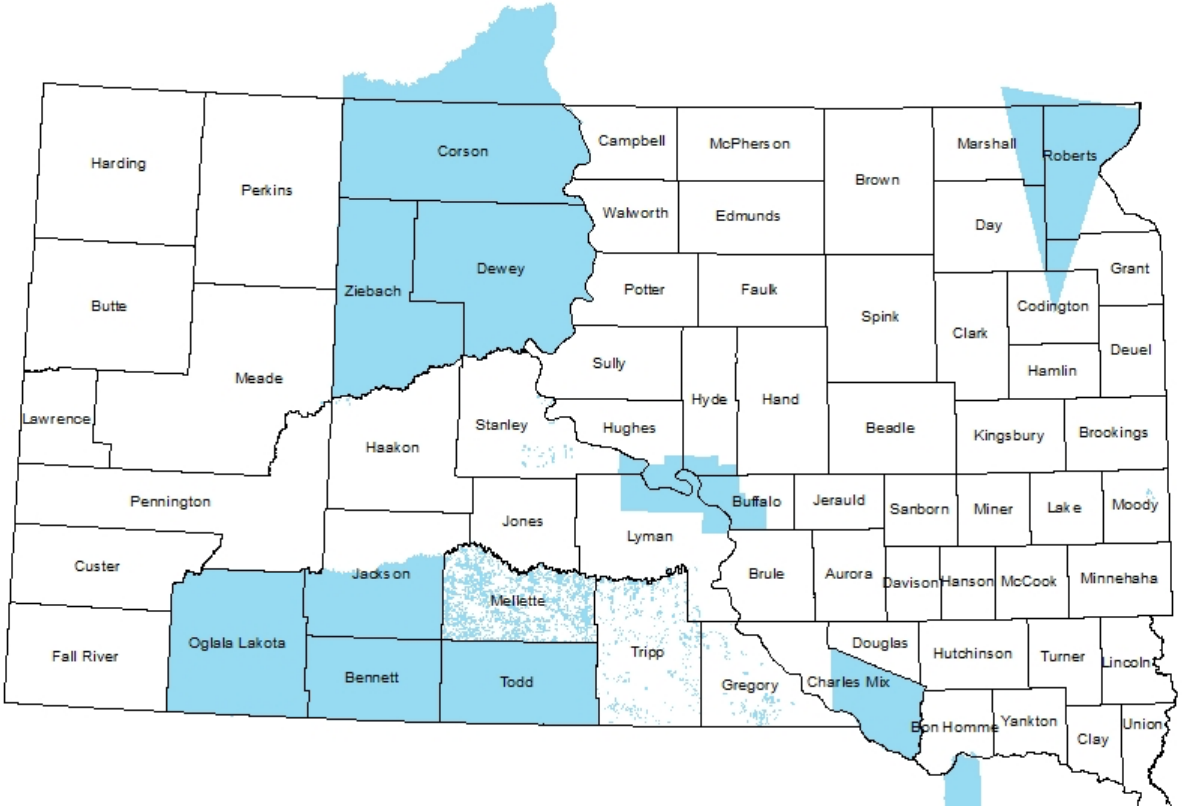

Note: Indian country is colored in blue. We adopted the federal definition of Indian Country, which includes federal Indian reservations and trust land allotments (18 USC § 1151; 40 CFR § 171.3), acknowledging that colloquially Indian Country encompasses a larger geographic area (National Congress of American Indians, 2019). Indian reservations are land reserved for a tribe or tribes under a treaty or other agreement with the federal government. Trust lands or allotments (referred to here as tribal lands) are lands where the title is held in trust by the United States for the benefit of American Indian tribes or individual American Indians. Indian Country makes up the entirety of six counties in South Dakota: Bennett, Corson, Dewey, Oglala Lakota, Todd, and Ziebach. Eighteen counties include both Indian and non-Indian Country with only one of those counties classified as urban. Of the 42 counties with no Indian Country, 35 are rural and 7 are urban (The United States Attorney's Office: District of South Dakota, 2022; United States Department of Agriculture). This map was created using shape files downloaded from the U.S. Department of the Interior (U.S. Department of the Interior: Indian Affairs), U.S. Census Bureau (U.S. Census Bureau, Department of Commerce, 2021), and the State of South Dakota (State of South Dakota, 2021).

eTable 1A: Demographic and criminal legal characteristics of convictions: Urban counties

	July 1, 2011 - June 30, 2013				July 1, 2013 – June 30, 2015			
	American Indian		White		American Indian		White	
	N	%	N	%	N	%	N	%
Penalty type								
Jail only	1,710	51.48	1,574	26.27	2,196	61.32	3,050	55.47
Jail and fine	1,612	48.52	4,417	73.73	1,385	38.68	2,448	44.53
Sex								
Male	2,216	66.71	4,296	71.71	2,418	67.52	3,928	71.44
Female	1,106	33.29	1,695	28.29	1,163	32.48	1,570	28.56
Age								
18-24	981	29.53	1,834	30.61	1,001	27.95	1,562	28.41
25-34	1,002	30.16	1,809	30.20	1,133	31.64	1,619	29.45
35-44	654	19.69	1,058	17.66	704	19.66	1,022	18.59
45+	685	20.62	1,290	21.53	743	20.75	1,295	23.55
Prior arrest								
0	456	13.73	2,007	33.50	441	12.31	1,798	32.70
1	319	9.60	1,110	18.53	351	9.80	983	17.88
2	294	8.85	676	11.28	298	8.32	626	11.39
3+	2,253	67.82	2,198	36.69	2,491	69.56	2,091	38.03
Charge type								
Drug	177	5.33	270	4.51	195	5.45	258	4.69
DUI	585	17.61	2,835	47.32	528	14.74	2,455	44.65
Property	766	23.06	1,070	17.86	975	27.23	1,092	19.86
Violent	347	10.45	739	12.34	467	13.04	807	14.68
Other	1,447	43.56	1,077	17.98	1,416	39.54	886	16.11
Jail sentence length (days)								
1-30	2,189	65.89	3,500	58.42	2,327	64.98	3,429	62.37
31-60	245	7.38	691	11.53	266	7.43	478	8.69
61-90	334	10.05	627	10.47	429	11.98	511	9.29
91+	554	16.68	1,173	19.58	559	15.61	1,080	19.64

eTable 1B: Demographic and criminal legal characteristics of convictions: Rural, no Indian Country counties

	July 1, 2011 - June 30, 2013				July 1, 2013 – June 30, 2015			
	American Indian		White		American Indian		White	
	N	%	N	%	N	%	N	%
Penalty type								
Jail only	269	30.78	643	14.32	461	53.92	1,738	43.15868
Jail and fine	605	69.22	3,847	85.68	394	46.08	2,289	56.84
Sex								
Male	529	60.53	3,308	73.67	491	57.43	2,901	72.04
Female	345	39.47	1,182	26.33	364	42.57	1,126	27.96
Age								
18-24	303	34.67	1,641	36.55	273	31.93	1,348	33.47
25-34	313	35.81	1,246	27.75	304	35.56	1,097	27.24
35-44	157	17.96	721	16.06	170	19.88	697	17.31
45+	101	11.56	882	19.64	108	12.63	885	21.98
Prior arrest								
0	216	24.71	1,906	42.45	159	18.60	1,537	38.17
1	138	15.79	812	18.08	118	13.80	730	18.13
2	103	11.78	506	11.27	103	12.05	436	10.83
3+	417	47.71	1,266	28.20	475	55.56	1,324	32.88
Charge type								
Drug	67	7.67	294	6.55	49	5.73	239	5.93
DUI	236	27.00	2,017	44.92	207	24.21	1,692	42.02
Property	156	17.85	621	13.83	173	20.23	702	17.43
Violent	245	28.03	781	17.39	264	30.88	725	18.00
Other	170	19.45	777	17.31	162	18.95	669	16.61
Jail sentence length (days)								
1-30	569	65.10	3,008	66.99	599	70.06	2,792	69.33
31-60	171	19.57	806	17.95	141	16.49	680	16.89
61-90	82	9.38	473	10.53	64	7.49	350	8.69
91+	52	5.95	203	4.52	51	5.96	205	5.09

eTable 1C: Demographic and criminal legal characteristics of convictions: Rural, Part Indian Country counties

	July 1, 2011 - June 30, 2013				July 1, 2013 – June 30, 2015			
	American Indian		White		American Indian		White	
	N	%	N	%	N	%	N	%
Penalty type								
Jail only	342	26.25	223	12.81	710	51.79	558	33.88
Jail and fine	961	73.75	1518	87.19	661	48.21	1089	66.12
Sex								
Male	798	61.24	1304	74.90	885	64.55	1254	76.14
Female	505	38.76	437	25.10	486	35.45	393	23.86
Age								
18-24	495	37.99	599	34.41	438	31.95	518	31.45
25-34	402	30.85	447	25.67	417	30.42	457	27.75
35-44	228	17.50	303	17.40	235	17.14	278	16.88
45+	178	13.66	392	22.52	281	20.50	394	23.92
Prior arrest								
0	308	23.64	707	40.61	243	17.72	694	42.14
1	210	16.12	346	19.87	176	12.84	323	19.61
2	143	10.97	201	11.55	158	11.52	179	10.87
3+	642	49.27	487	27.97	794	57.91	451	27.38
Charge type								
Drug	72	5.53	154	8.85	109	7.95	169	10.26
DUI	422	32.39	944	54.22	385	28.08	904	54.89
Property	275	21.11	158	9.08	208	15.17	131	7.95
Violent	208	15.96	243	13.96	230	16.78	242	14.69
Other	326	25.02	242	13.90	439	32.02	201	12.20
Jail sentence length (days)								
1-30	751	57.64	1,153	66.23	857	62.51	1,133	68.79
31-60	312	23.94	323	18.55	319	23.27	253	15.36
61-90	184	14.12	200	11.49	113	8.24	192	11.66
91+	56	4.30	65	3.73	82	5.98	69	4.19

eFigure 3A: Percent of convictions in urban counties assessed a fine: Urban counties

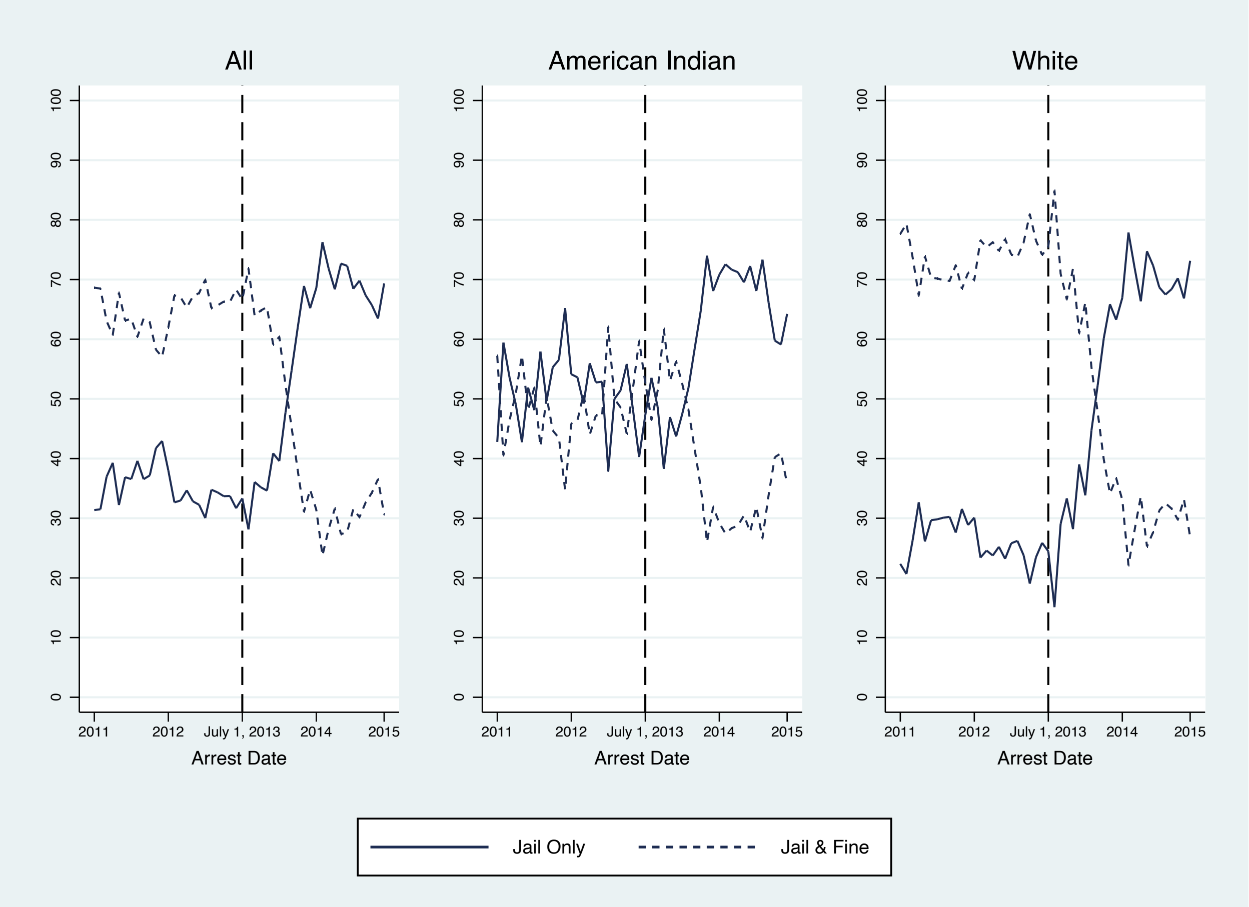

Note: The figures depict the percent of misdemeanor convictions assessed a fine two years before and after the reform's July 1, 2013 effective date.

eFigure 3B: Percent of convictions in rural, no Indian Country counties assessed a fine: Rural, No Indian Country counties

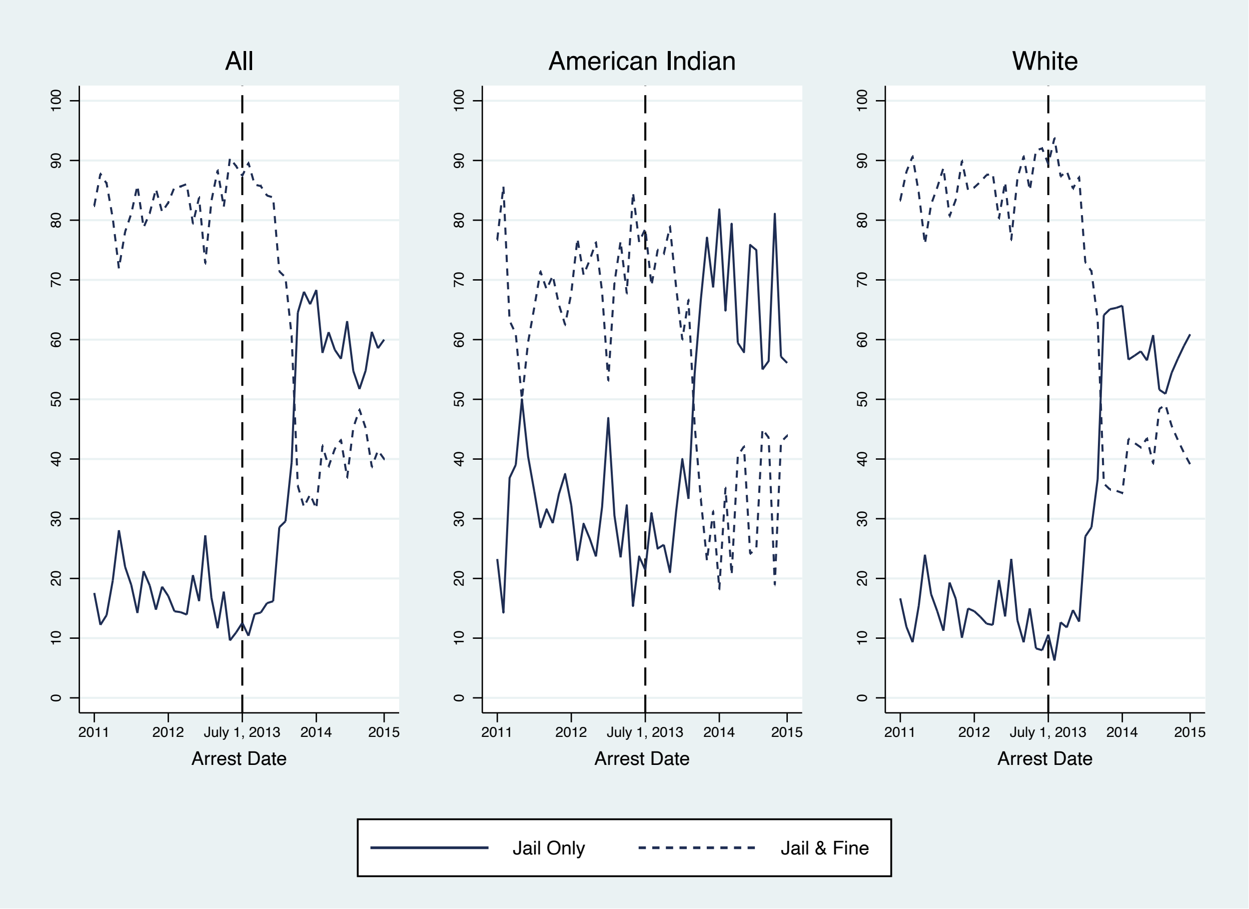

Note: The figures depict the percent of misdemeanor convictions assessed a fine two years before and after the reform's July 1, 2013 effective date.

eFigure 3C: Percent of convictions in rural, part Indian Country counties assessed a fine: Rural, Part Indian Country counties

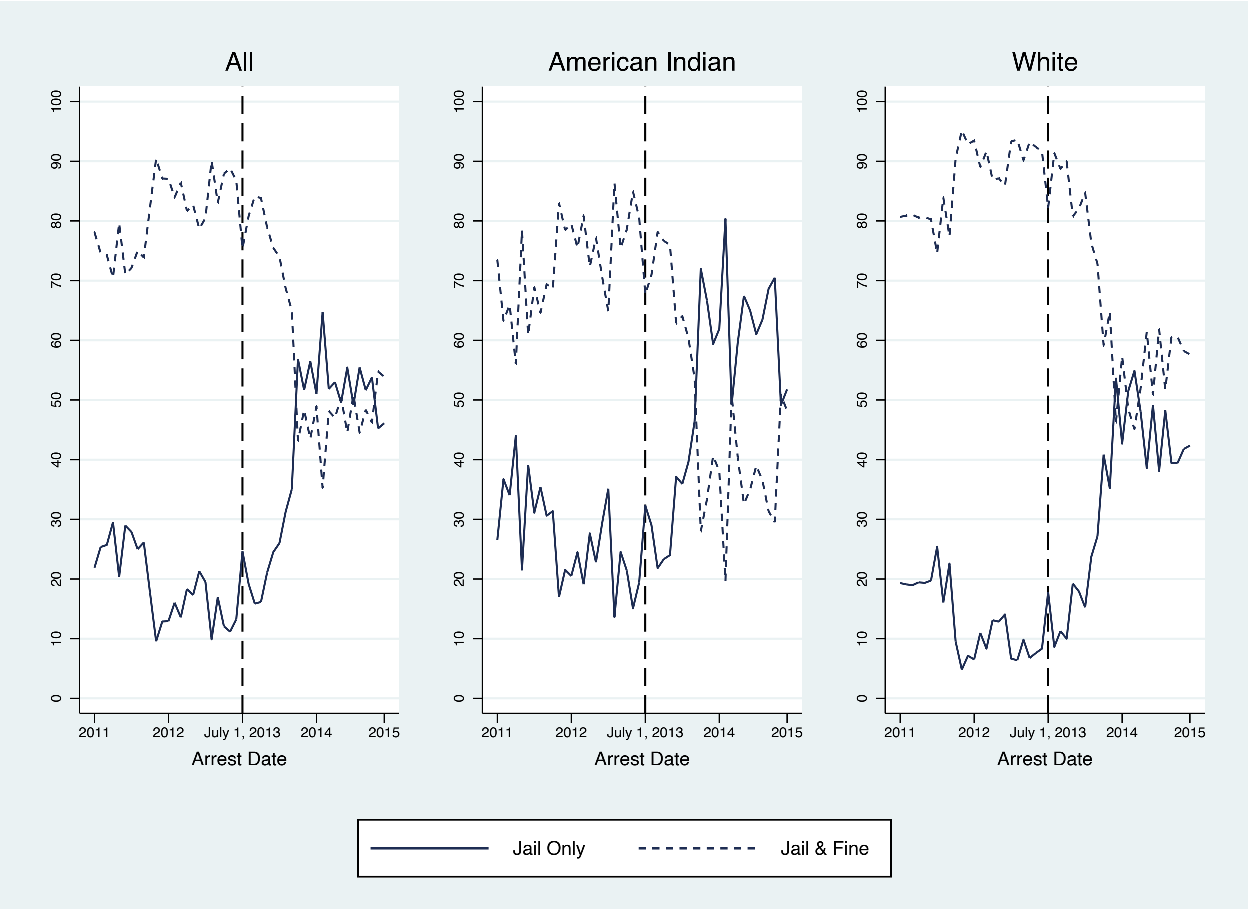

Note: The figures depict the percent of misdemeanor convictions assessed a fine two years before and after the reform's July 1, 2013 effective date.

eTable 2A: Logistic regression models examining association between binary reform indicator and fine assessment

	Urban	Rural, No Indian Country	Rural, Part Indian Country
Variables	OR (95% CI)	OR (95% CI)	OR (95% CI)
Reform (ref. pre-reform)	0.63 (0.39 - 1.04)	0.24** (0.18 - 0.33)	0.24** (0.18 - 0.32)
Unemployment rate	1.99** (1.19 - 3.33)	1.28 (0.93 - 1.75)	0.93 (0.72 - 1.19)
Male (ref. female)	0.98 (0.91 - 1.06)	1.08 (0.98 - 1.20)	1.26** (1.09 - 1.46)
Age (ref. 18 - <25 years)			
25 - <35	0.99 (0.90 - 1.08)	0.95 (0.84 - 1.07)	0.99 (0.83 - 1.17)
35 - <45	1.00 (0.90 - 1.11)	0.96 (0.83 - 1.11)	0.96 (0.79 - 1.16)
45+	0.82** (0.73 - 0.92)	1.05 (0.91 - 1.21)	1.14 (0.95 - 1.35)
Prior arrest (ref. no prior)			
1	0.79** (0.70 - 0.89)	1.01 (0.88 - 1.16)	0.99 (0.83 - 1.19)
2	0.77** (0.68 - 0.87)	0.83* (0.71 - 0.98)	0.82 (0.66 - 1.02)
3	0.65** (0.57 - 0.73)	0.76** (0.66 - 0.87)	0.71** (0.59 - 0.86)
Drug charge	1.93** (1.57 - 2.37)	1.02 (0.79 - 1.31)	1.31 (0.98 - 1.76)
Property charge	1.46** (1.28 - 1.65)	0.72** (0.59 - 0.89)	1.15 (0.94 - 1.40)
Violent charge	0.85* (0.74 - 0.98)	1.08 (0.91 - 1.28)	0.71** (0.56 - 0.89)
DUI charge	2.82** (2.24 - 3.55)	1.46** (1.22 - 1.73)	2.30** (1.81 - 2.91)
Jail sentence length (ref. 1–30 days)			
31 – 60	0.56** (0.48 - 0.66)	0.69** (0.58 - 0.82)	0.65** (0.55 - 0.78)
61 – 90	0.77** (0.68 - 0.88)	0.70** (0.56 - 0.87)	0.72* (0.56 - 0.94)
91+	0.32** (0.29 - 0.37)	0.66** (0.52 - 0.82)	0.38** (0.29 - 0.51)
Observations	18,392	10,225	6,061

Notes: ** p<0.01, * p<0.05. The control variables for all models include gender, age, prior arrests, jail sentence length, charge type, and county unemployment, as well as county and month fixed effects. All standard errors are clustered by county and month-year. The periods for the binary indicator are pre-reform (July 1, 2011 – June 30, 2013) and post-reform (July 1, 2013 – June 30, 2015).

eTable 2B: Logistic regression models examining association between 6-month interval reform indicator and fine assessment

	Urban	Rural, No Indian Country	Rural, Part Indian Country
Variables	OR (95% CI)	OR (95% CI)	OR (95% CI)
Reform (ref. pre-reform)			
6-months post	0.79 (0.53 - 1.17)	1.19 (0.86 - 1.65)	0.76 (0.52 - 1.11)
12-months post	0.31** (0.20 - 0.47)	0.14** (0.09 - 0.20)	0.17** (0.11 - 0.26)
18-months post	0.12** (0.07 - 0.20)	0.08** (0.06 - 0.12)	0.13** (0.09 - 0.19)
24-months post	0.18** (0.10 - 0.31)	0.09** (0.06 - 0.13)	0.12** (0.08 - 0.19)
Unemployment rate	0.70 (0.46 - 1.07)	0.76* (0.58 - 0.99)	0.76* (0.60 - 0.96)
Male (ref. female)	0.98 (0.91 - 1.06)	1.09 (0.99 - 1.21)	1.30** (1.12 - 1.51)
Age (ref. 18 - <25 years)			
25 - <35	1.00 (0.91 - 1.09)	0.99 (0.87 - 1.13)	0.97 (0.81 - 1.16)
35 - <45	1.02 (0.91 - 1.13)	1.04 (0.89 - 1.21)	0.95 (0.78 - 1.16)
45+	0.82** (0.73 - 0.92)	1.11 (0.96 - 1.29)	1.12 (0.93 - 1.33)
Prior arrest (ref. no prior)			
1	0.78** (0.69 - 0.88)	0.99 (0.86 - 1.15)	1.00 (0.83 - 1.21)
2	0.76** (0.68 - 0.86)	0.79** (0.67 - 0.94)	0.78* (0.63 - 0.97)
3	0.64** (0.57 - 0.72)	0.74** (0.64 - 0.85)	0.72** (0.59 - 0.86)
Drug charge	2.01** (1.63 - 2.47)	1.04 (0.80 - 1.35)	1.40* (1.03 - 1.89)
Property charge	1.50** (1.32 - 1.71)	0.72** (0.58 - 0.90)	1.13 (0.91 - 1.40)
Violent charge	0.85* (0.74 - 0.98)	1.02 (0.85 - 1.22)	0.68** (0.53 - 0.87)
DUI charge	2.94** (2.33 - 3.71)	1.45** (1.20 - 1.75)	2.31** (1.81 - 2.95)
Jail sentence length (ref. 1–30 days)			
31 – 60	0.55** (0.47 - 0.64)	0.63** (0.52 - 0.76)	0.65** (0.54 - 0.78)
61 – 90	0.73** (0.64 - 0.84)	0.62** (0.49 - 0.78)	0.74* (0.57 - 0.97)
91+	0.31** (0.27 - 0.35)	0.60** (0.47 - 0.76)	0.38** (0.28 - 0.51)
Observations	18,392	10,225	6,061

Notes: ** $p<0.01$, * $p<0.05$. The control variables for all models include gender, age, prior arrests, jail sentence length, charge type, and county unemployment, as well as county and month fixed effects. All standard errors are clustered by county and month-year. The periods for the 6-month interval indicator refer to the following dates: pre- reform: July 1, 2011 – June 30, 2013; 6-months post: July 1, 2013-December 31, 2013; 12-months post: January 1, 2014 – June 30, 2014; 18-months post: July 1, 2014 – December 31, 2014; 24-months post: January 1, 2015 – June 30, 2015.

eTable 2C: Marginal effect estimates of reform on fine assessment

	Urban	Rural, No Indian Country	Rural, Part Indian Country
Panel A: Binary reform indicator (ref. pre-reform)			
Post-reform	-0.10 (-0.21 – 0.01)	-0.25** (-0.31 - -0.20)	-0.24** (-0.29 - -0.20)
Panel B: 6-month interval reform indicator (ref. pre-reform)			
6-months post	-0.05 (-0.13 - 0.03)	0.02 (-0.02 - 0.06)	-0.04 (-0.09 - 0.02)
12-months post	-0.25** (-0.34 - -0.16)	-0.36** (-0.43 - -0.29)	-0.31** (-0.38 - -0.23)
18-months post	-0.43** (-0.53 - -0.34)	-0.46** (-0.52 - -0.41)	-0.36** (-0.42 - -0.30)
24-months post	-0.36** (-0.47 - -0.26)	-0.45** (-0.53 - -0.38)	-0.37** (-0.45 - -0.29)
Observations	18,392	10,225	6,061

Notes: ** $p<0.01$, * $p<0.05$. The control variables for all models include gender, age, prior arrests, jail sentence length, charge type, and county unemployment, as well as county and month fixed effects. All standard errors are clustered by county and month-year. The periods for the binary indicator are pre-reform (July 1, 2011 – June 30, 2013) and post-reform (July 1, 2013 – June 30, 2015). The periods for the 6-month interval indicator refer to the following dates: pre-reform: July 1, 2011 – June 30, 2013; 6-months post: July 1, 2013-December 31, 2013; 12-months post: January 1, 2014 – June 30, 2014; 18-months post: July 1, 2014 – December 31, 2014; 24-months post: January 1, 2015 – June 30, 2015.

eTable 3A: Logistic regression models examining association between binary reform indicator, race, and fine assessment without American Indian main effect

Variables	Urban	Rural, No Indian Country	Rural, Part Indian Country
	OR (95% CI)	OR (95% CI)	OR (95% CI)
Reform (ref. pre-reform)	0.45** (0.27 - 0.73)	0.22** (0.16 - 0.29)	0.22** (0.15 - 0.31)
Reform*AI (ref. White)	1.22* (1.02 - 1.44)	0.86 (0.73 - 1.02)	0.71** (0.57 - 0.88)
Unemployment rate	2.02** (1.23 - 3.32)	1.29 (0.95 - 1.76)	0.93 (0.72 - 1.18)
Male (ref. female)	0.96 (0.89 - 1.03)	1.04 (0.94 - 1.15)	1.18* (1.02 - 1.36)
Age (ref. 18 - <25 years)			
25 - <35	0.98 (0.89 - 1.07)	0.95 (0.84 - 1.08)	0.96 (0.81 - 1.14)
35 - <45	0.99 (0.89 - 1.10)	0.95 (0.82 - 1.10)	0.91 (0.75 - 1.11)
45+	0.81** (0.72 - 0.91)	1.02 (0.88 - 1.17)	1.06 (0.89 - 1.26)
Prior arrest (ref. no prior)			
1	0.79** (0.70 - 0.89)	1.03 (0.90 - 1.19)	1.02 (0.85 - 1.22)
2	0.78** (0.68 - 0.88)	0.85 (0.72 - 1.00)	0.86 (0.69 - 1.07)
3	0.68** (0.61 - 0.76)	0.80** (0.69 - 0.92)	0.79* (0.65 - 0.95)
Drug charge	1.85** (1.50 - 2.28)	1.02 (0.80 - 1.31)	1.26 (0.94 - 1.69)
Property charge	1.40** (1.23 - 1.59)	0.73** (0.60 - 0.89)	1.17 (0.95 - 1.43)
Violent charge	0.80** (0.69 - 0.93)	1.12 (0.94 - 1.33)	0.71** (0.56 - 0.90)
DUI charge	2.65** (2.11 - 3.32)	1.43** (1.20 - 1.71)	2.19** (1.72 - 2.79)
Jail sentence length (ref. 1–30 days)			
31 – 60	0.55** (0.47 - 0.64)	0.70** (0.58 - 0.83)	0.67** (0.56 - 0.80)
61 – 90	0.75** (0.66 - 0.86)	0.69** (0.55 - 0.87)	0.73* (0.56 - 0.95)
91+	0.32** (0.28 - 0.36)	0.66** (0.53 - 0.83)	0.38** (0.29 - 0.51)
Observations	18,392	10,225	6,061

Notes: ** p<0.01, * p<0.05. AI refers to American Indian. AI equals 1 if the convicted person's race was recorded as American Indian and 0 if the recorded race was White. The control variables for all models include gender, age, prior arrests, jail sentence length, charge type, and county unemployment, as well as county and month fixed effects. All standard errors are clustered by county and month-year. The periods for the binary indicator are pre-reform (July 1, 2011 – June 30, 2013) and post-reform (July 1, 2013 – June 30, 2015).

eTable 3B: Logistic regression models examining association between 6-month interval reform indicator, race, and fine assessment without American Indian independent effect

Variables	Urban	Rural, No Indian Country	Rural, Part Indian Country
	OR (95% CI)	OR (95% CI)	OR (95% CI)
Reform (ref. pre-reform)			
6-months post	0.73 (0.48 - 1.11)	1.23 (0.88 - 1.73)	0.78 (0.49 - 1.25)
12-months post	0.21** (0.13 - 0.33)	0.12** (0.08 - 0.17)	0.15** (0.09 - 0.23)
18-months post	0.08** (0.04 - 0.13)	0.07** (0.05 - 0.10)	0.12** (0.07 - 0.18)
24-months post	0.10** (0.06 - 0.17)	0.07** (0.04 - 0.11)	0.11** (0.07 - 0.19)
Reform*AI (ref. White)			
6-months post	0.60* (0.40 - 0.91)	0.44** (0.29 - 0.68)	0.55* (0.35 - 0.87)
12-months post	1.18 (0.85 - 1.64)	0.86 (0.61 - 1.19)	0.77 (0.52 - 1.15)
18-months post	1.65** (1.19 - 2.29)	0.88 (0.58 - 1.32)	0.72 (0.49 - 1.06)
24-months post	1.97** (1.41 - 2.74)	1.13 (0.80 - 1.59)	0.68* (0.46 - 1.00)
Unemployment rate	0.69 (0.47 - 1.01)	0.75* (0.58 - 0.97)	0.76* (0.60 - 0.96)
Male (ref. female)	0.95 (0.88 - 1.03)	1.04 (0.94 - 1.16)	1.21* (1.04 - 1.40)
Age (ref. 18 - <25 years)			
25 - <35	0.98 (0.90 - 1.08)	0.99 (0.87 - 1.14)	0.94 (0.79 - 1.13)
35 - <45	1.01 (0.91 - 1.13)	1.03 (0.88 - 1.20)	0.91 (0.74 - 1.11)
45+	0.81** (0.72 - 0.91)	1.07 (0.92 - 1.24)	1.04 (0.87 - 1.24)
Prior arrest (ref. no prior)			
1	0.78** (0.69 - 0.88)	1.01 (0.87 - 1.18)	1.03 (0.86 - 1.24)
2	0.77** (0.68 - 0.87)	0.81* (0.69 - 0.97)	0.82 (0.66 - 1.03)
3	0.67** (0.60 - 0.75)	0.78** (0.68 - 0.91)	0.80* (0.66 - 0.96)
Drug charge	1.93** (1.56 - 2.39)	1.05 (0.81 - 1.36)	1.34 (0.99 - 1.82)
Property charge	1.44** (1.27 - 1.62)	0.72** (0.58 - 0.90)	1.15 (0.93 - 1.43)
Violent charge	0.80** (0.69 - 0.92)	1.06 (0.89 - 1.27)	0.68** (0.53 - 0.87)
DUI charge	2.76** (2.20 - 3.47)	1.42** (1.18 - 1.72)	2.20** (1.71 - 2.83)
Jail sentence length (ref. 1–30 days)			
31 – 60	0.54** (0.46 - 0.62)	0.64** (0.53 - 0.76)	0.66** (0.55 - 0.79)
61 – 90	0.71** (0.62 - 0.81)	0.62** (0.49 - 0.78)	0.75* (0.57 - 0.98)
91+	0.30** (0.27 - 0.34)	0.61** (0.48 - 0.78)	0.38** (0.28 - 0.52)
Observations	18,392	10,225	6,061

Notes: ** p<0.01, * p<0.05. AI refers to American Indian. AI equals 1 if the convicted person's race was recorded as American Indian and 0 if the recorded race was White. The control variables for all models include gender, age, prior arrests, jail sentence length, charge type, and county unemployment, as well as county and month fixed effects. All standard errors are clustered by county and month-year. The periods for the 6-month interval indicator refer to the following dates: pre- reform: July 1, 2011 – June 30, 2013; 6-months post: July 1, 2013-December 31, 2013; 12-months post: January 1, 2014 – June 30, 2014; 18-months post: July 1, 2014 – December 31, 2014; 24-months post: January 1, 2015 – June 30, 2015.

eTable 3C: Marginal effect estimates of American Indian (Ref. White) on fine assessment before and after reform

	Urban	Rural, No Indian Country	Rural, Part Indian Country
Panel A: Binary reform indicator			
Before	-0.15** (-0.18 - -0.12)	-0.11** (-0.15 - -0.08)	-0.07** (-0.11 - -0.04)
After	0.04* (0.01 - 0.08)	-0.03 (-0.07 - 0.00)	-0.07** (-0.11 - -0.02)
Panel B: 6-month interval reform indicator			
Pre-reform	-0.14** (-0.17 - -0.11)	-0.11** (-0.14 - -0.07)	-0.07** (-0.10 - -0.04)
6-months post	-0.11* (-0.19 - -0.02)	-0.11** (-0.17 - -0.04)	-0.08* (-0.15 - -0.02)
12-months post	0.04 (-0.03 - 0.11)	-0.03 (-0.10 - 0.04)	-0.05 (-0.14 - 0.03)
18-months post	0.09** (0.03 - 0.14)	-0.02 (-0.10 - 0.05)	-0.07 (-0.15 - 0.01)
24-months post	0.13** (0.07 - 0.20)	0.02 (-0.04 - 0.09)	-0.08* (-0.16 - -0.00)
Observations	18,392	10,225	6,061

Notes: ** $p<0.01$, * $p<0.05$. The control variables for all models include gender, age, prior arrests, jail sentence length, charge type, and county unemployment, as well as county and month fixed effects. All standard errors are clustered by county and month-year. The periods for the binary indicator are pre-reform (July 1, 2011 – June 30, 2013) and post-reform (July 1, 2013 – June 30, 2015). The periods for the 6-month interval indicator refer to the following dates: pre-reform: July 1, 2011 – June 30, 2013; 6-months post: July 1, 2013-December 31, 2013; 12-months post: January 1, 2014 – June 30, 2014; 18-months post: July 1, 2014 – December 31, 2014; 24-months post: January 1, 2015 – June 30, 2015.

eTable 4A: Logistic regression models examining association between binary reform indicator, race, and fine assessment with American Indian main effect

	Urban	Rural, No Indian Country	Rural, Part Indian Country
VARIABLES	OR (95% CI)	OR (95% CI)	OR (95% CI)
Reform (ref. pre-reform)	0.45** (0.27 - 0.73)	0.22** (0.16 - 0.29)	0.22** (0.15 - 0.31)
AI (ref. White)	0.49** (0.42 - 0.57)	0.47** (0.38 - 0.58)	0.57** (0.45 - 0.73)
Reform*AI	2.48** (1.96 - 3.14)	1.83** (1.38 - 2.42)	1.25 (0.87 - 1.78)
Unemployment rate	2.02** (1.23 - 3.32)	1.29 (0.95 - 1.76)	0.93 (0.72 - 1.18)
Male (ref. female)	0.96 (0.89 - 1.03)	1.04 (0.94 - 1.15)	1.18* (1.02 - 1.36)
Age (ref. 18 - <25 years)			
25 - <35	0.98 (0.89 - 1.07)	0.95 (0.84 - 1.08)	0.96 (0.81 - 1.14)
35 - <45	0.99 (0.89 - 1.10)	0.95 (0.82 - 1.10)	0.91 (0.75 - 1.11)
45+	0.81** (0.72 - 0.91)	1.02 (0.88 - 1.17)	1.06 (0.89 - 1.26)
Prior arrest (ref. no prior)			
1	0.79** (0.70 - 0.89)	1.03 (0.90 - 1.19)	1.02 (0.85 - 1.22)
2	0.78** (0.68 - 0.88)	0.85 (0.72 - 1.00)	0.86 (0.69 - 1.07)
3	0.68** (0.61 - 0.76)	0.80** (0.69 - 0.92)	0.79* (0.65 - 0.95)
Drug charge	1.85** (1.50 - 2.28)	1.02 (0.80 - 1.31)	1.26 (0.94 - 1.69)
Property charge	1.40** (1.23 - 1.59)	0.73** (0.60 - 0.89)	1.17 (0.95 - 1.43)
Violent charge	0.80** (0.69 - 0.93)	1.12 (0.94 - 1.33)	0.71** (0.56 - 0.90)
DUI charge	2.65** (2.11 - 3.32)	1.43** (1.20 - 1.71)	2.19** (1.72 - 2.79)
Jail sentence length (ref. 1–30 days)			
31 – 60	0.55** (0.47 - 0.64)	0.70** (0.58 - 0.83)	0.67** (0.56 - 0.80)
61 – 90	0.75** (0.66 - 0.86)	0.69** (0.55 - 0.87)	0.73* (0.56 - 0.95)
91+	0.32** (0.28 - 0.36)	0.66** (0.53 - 0.83)	0.38** (0.29 - 0.51)
Observations	18,392	10,225	6,061

Notes: ** p<0.01, * p<0.05. AI refers to American Indian. AI equals 1 if the convicted person's race was recorded as American Indian and 0 if the recorded race was White. The control variables for all models include gender, age, prior arrests, jail sentence length, charge type, and county unemployment, as well as county and month fixed effects. All standard errors are clustered by county and month-year. The periods for the binary indicator are pre-reform (July 1, 2011 – June 30, 2013) and post-reform (July 1, 2013 – June 30, 2015).

eTable 4B: Logistic regression models examining association between 6-month interval reform indicator, race, and fine assessment with American Indian independent effect

	Urban	Rural, No Indian Country	Rural, Part Indian Country
Variables	OR (95% CI)	OR (95% CI)	OR (95% CI)
Reform (ref. pre-reform)			
6-months post	0.73 (0.48 - 1.11)	1.23 (0.88 - 1.73)	0.78 (0.49 - 1.25)
12-months post	0.21** (0.13 - 0.33)	0.12** (0.08 - 0.17)	0.15** (0.09 - 0.23)
18-months post	0.08** (0.04 - 0.13)	0.07** (0.05 - 0.10)	0.12** (0.07 - 0.18)
24-months post	0.10** (0.06 - 0.17)	0.07** (0.04 - 0.11)	0.11** (0.07 - 0.19)
AI (ref. white)	0.49** (0.43 - 0.56)	0.47** (0.38 - 0.57)	0.58** (0.46 - 0.74)
Reform*AI (ref. White)			
6-months post	1.22 (0.79 - 1.89)	0.94 (0.59 - 1.52)	0.96 (0.57 - 1.61)
12-months post	2.41** (1.71 - 3.40)	1.82** (1.24 - 2.67)	1.33 (0.82 - 2.17)
18-months post	3.37** (2.39 - 4.75)	1.87** (1.18 - 2.96)	1.24 (0.78 - 1.97)
24-months post	4.01** (2.83 - 5.68)	2.40** (1.58 - 3.65)	1.17 (0.73 - 1.87)
Unemployment rate	0.69 (0.47 - 1.01)	0.75* (0.58 - 0.97)	0.76* (0.60 - 0.96)
Male (ref. female)	0.95 (0.88 - 1.03)	1.04 (0.94 - 1.16)	1.21* (1.04 - 1.40)
Age (ref. 18 - <25 years)			
25 - <35	0.98 (0.90 - 1.08)	0.99 (0.87 - 1.14)	0.94 (0.79 - 1.13)
35 - <45	1.01 (0.91 - 1.13)	1.03 (0.88 - 1.20)	0.91 (0.74 - 1.11)
45+	0.81** (0.72 - 0.91)	1.07 (0.92 - 1.24)	1.04 (0.87 - 1.24)
Prior arrest (ref. no prior)			
1	0.78** (0.69 - 0.88)	1.01 (0.87 - 1.18)	1.03 (0.86 - 1.24)
2	0.77** (0.68 - 0.87)	0.81* (0.69 - 0.97)	0.82 (0.66 - 1.03)
3	0.67** (0.60 - 0.75)	0.78** (0.68 - 0.91)	0.80* (0.66 - 0.96)
Drug charge	1.93** (1.56 - 2.39)	1.05 (0.81 - 1.36)	1.34 (0.99 - 1.82)
Property charge	1.44** (1.27 - 1.62)	0.72** (0.58 - 0.90)	1.15 (0.93 - 1.43)
Violent charge	0.80** (0.69 - 0.92)	1.06 (0.89 - 1.27)	0.68** (0.53 - 0.87)
DUI charge	2.76** (2.20 - 3.47)	1.42** (1.18 - 1.72)	2.20** (1.71 - 2.83)
Jail sentence length (ref. 1–30 days)			
31 – 60	0.54** (0.46 - 0.62)	0.64** (0.53 - 0.76)	0.66** (0.55 - 0.79)
61 – 90	0.71** (0.62 - 0.81)	0.62** (0.49 - 0.78)	0.75* (0.57 - 0.98)
91+	0.30** (0.27 - 0.34)	0.61** (0.48 - 0.78)	0.38** (0.28 - 0.52)
Observations	18,392	10,225	6,061

Notes: ** p<0.01, * p<0.05. AI refers to American Indian. AI equals 1 if the convicted person's race was recorded as American Indian and 0 if the recorded race was White. The control variables for all models include gender, age, prior arrests, jail sentence length, charge type, and county unemployment, as well as county and month fixed effects. All standard errors are clustered by county and month-year. The periods for the 6-month interval indicator refer to the following dates: pre- reform: July 1, 2011 – June 30, 2013; 6-months post: July 1, 2013- December 31, 2013; 12-months post: January 1, 2014 – June 30, 2014; 18-months post: July 1, 2014 – December 31, 2014; 24-months post: January 1, 2015 – June 30, 2015.

eTable 4C: Logistic regression models examining association between binary reform indicator and fine assessment including all persons regardless of recorded race

	Urban	Rural, No Indian Country	Rural, Part Indian Country
Variables	OR (95% CI)	OR (95% CI)	OR (95% CI)
Reform (ref. pre-reform)	0.60 (0.36 - 1.01)	0.25** (0.18 - 0.33)	0.24** (0.18 - 0.33)
Unemployment rate	1.93* (1.14 - 3.28)	1.33 (0.97 - 1.82)	0.96 (0.75 - 1.23)
Male (ref. female)	0.98 (0.92 - 1.05)	1.07 (0.97 - 1.18)	1.25** (1.09 - 1.44)
Age (ref. 18 - <25 years)			
25 - <35	0.99 (0.91 - 1.07)	0.96 (0.85 - 1.08)	0.96 (0.81 - 1.13)
35 - <45	0.99 (0.90 - 1.09)	0.97 (0.84 - 1.11)	0.95 (0.79 - 1.14)
45+	0.82** (0.73 - 0.92)	1.07 (0.93 - 1.23)	1.13 (0.95 - 1.35)
Prior arrest (ref. no prior)			
1	0.82** (0.73 - 0.92)	1.00 (0.88 - 1.15)	1.01 (0.85 - 1.21)
2	0.78** (0.69 - 0.87)	0.81** (0.69 - 0.95)	0.82 (0.67 - 1.02)
3	0.65** (0.58 - 0.72)	0.74** (0.65 - 0.85)	0.73** (0.61 - 0.87)
Drug charge	1.96** (1.59 - 2.41)	1.06 (0.84 - 1.34)	1.30 (0.98 - 1.72)
Property charge	1.43** (1.27 - 1.61)	0.74** (0.61 - 0.90)	1.17 (0.96 - 1.42)
Violent charge	0.85* (0.74 - 0.97)	1.07 (0.91 - 1.25)	0.70** (0.56 - 0.88)
DUI charge	2.59** (2.06 - 3.25)	1.45** (1.23 - 1.71)	2.34** (1.86 - 2.95)
Jail sentence length (ref. 1–30 days)			
31 – 60	0.57** (0.49 - 0.66)	0.70** (0.60 - 0.82)	0.67** (0.56 - 0.80)
61 – 90	0.75** (0.66 - 0.85)	0.75** (0.61 - 0.93)	0.75* (0.58 - 0.97)
91+	0.33** (0.29 - 0.37)	0.70** (0.56 - 0.87)	0.37** (0.28 - 0.49)
Observations	20,666	11,398	6,384

Notes: ** p<0.01, * p<0.05. Models do not restrict convictions to those associated with persons whose recorded race was American Indian or White, including all individuals in the dataset regardless of recorded race. The control variables for all models include gender, age, prior arrests, jail sentence length, charge type, and county unemployment, as well as county and month fixed effects. All standard errors are clustered by county and month-year. The periods for the binary indicator are pre-reform (July 1, 2011 – June 30, 2013) and post-reform (July 1, 2013 – June 30, 2015).

eTable 4D: Logistic regression models examining association between 6-month interval reform indicator and fine assessment including all persons regardless of recorded race

Variables	Urban	Rural, No Indian Country	Rural, Part Indian Country
	OR (95% CI)	OR (95% CI)	OR (95% CI)
Reform (ref. pre-reform)			
6-months post	0.75 (0.50 - 1.13)	1.20 (0.87 - 1.66)	0.82 (0.56 - 1.20)
12-months post	0.29** (0.19 - 0.45)	0.13** (0.09 - 0.20)	0.17** (0.11 - 0.26)
18-months post	0.11** (0.07 - 0.19)	0.08** (0.05 - 0.11)	0.13** (0.09 - 0.19)
24-months post	0.16** (0.09 - 0.28)	0.08** (0.05 - 0.13)	0.12** (0.08 - 0.19)
Unemployment rate	0.66 (0.43 - 1.02)	0.76* (0.59 - 0.99)	0.77* (0.61 - 0.98)
Male (ref. female)	0.99 (0.92 - 1.06)	1.07 (0.97 - 1.19)	1.29** (1.12 - 1.49)
Age (ref. 18 - <25 years)			
25 - <35	1.00 (0.91 - 1.08)	1.00 (0.88 - 1.13)	0.94 (0.79 - 1.13)
35 - <45	1.01 (0.92 - 1.11)	1.04 (0.90 - 1.21)	0.94 (0.78 - 1.14)
45+	0.83** (0.74 - 0.92)	1.13 (0.98 - 1.31)	1.12 (0.94 - 1.33)
Prior arrest (ref. no prior)			
1	0.81** (0.72 - 0.91)	0.99 (0.86 - 1.14)	1.02 (0.85 - 1.23)
2	0.78** (0.69 - 0.87)	0.77** (0.65 - 0.91)	0.79* (0.63 - 0.98)
3	0.64** (0.57 - 0.71)	0.72** (0.63 - 0.83)	0.73** (0.61 - 0.88)
Drug charge	2.02** (1.64 - 2.49)	1.09 (0.85 - 1.39)	1.38* (1.03 - 1.85)
Property charge	1.47** (1.31 - 1.66)	0.73** (0.59 - 0.90)	1.14 (0.93 - 1.41)
Violent charge	0.85* (0.74 - 0.96)	1.00 (0.85 - 1.19)	0.67** (0.53 - 0.85)
DUI charge	2.69** (2.14 - 3.39)	1.45** (1.21 - 1.74)	2.37** (1.86 - 3.00)
Jail sentence length (ref. 1–30 days)			
31 – 60	0.56** (0.49 - 0.64)	0.64** (0.54 - 0.76)	0.66** (0.55 - 0.79)
61 – 90	0.71** (0.63 - 0.81)	0.67** (0.54 - 0.83)	0.77* (0.59 - 0.99)
91+	0.31** (0.28 - 0.35)	0.63** (0.50 - 0.79)	0.37** (0.27 - 0.49)
Observations	20,666	11,398	6,384

Notes: ** p<0.01, * p<0.05. Models do not restrict convictions to those associated with persons whose recorded race was American Indian or White, including all individuals in the dataset regardless of recorded race. The control variables for all models include gender, age, prior arrests, jail sentence length, charge type, and county unemployment, as well as county and month fixed effects. All standard errors are clustered by county and month-year. The periods for the 6-month interval indicator refer to the following dates: pre- reform: July 1, 2011 – June 30, 2013; 6-months post: July 1, 2013-December 31, 2013; 12-months post: January 1, 2014 – June 30, 2014; 18-months post: July 1, 2014 – December 31, 2014; 24-months post: January 1, 2015 – June 30, 2015.

eTable 4E: Logistic regression models examining association between binary reform indicator, race, and fine assessment including all persons regardless of recorded race

Variables	Urban	Rural, No Indian Country	Rural, Part Indian Country
	OR (95% CI)	OR (95% CI)	OR (95% CI)
Reform (ref. pre-reform)	0.44** (0.26 - 0.73)	0.22** (0.16 - 0.30)	0.22** (0.15 - 0.32)
Reform*race (ref. White)			
AI	1.19* (1.00 - 1.41)	0.87 (0.74 - 1.03)	0.72** (0.58 - 0.90)
Other	1.03 (0.88 - 1.19)	0.93 (0.76 - 1.14)	0.59** (0.40 - 0.86)
Unemployment rate	1.95* (1.16 - 3.26)	1.34 (0.98 - 1.83)	0.96 (0.75 - 1.23)
Male (ref. female)	0.96 (0.89 - 1.03)	1.03 (0.93 - 1.14)	1.19* (1.03 - 1.37)
Age (ref. 18 - <25 years)			
25 - <35	0.98 (0.90 - 1.06)	0.96 (0.85 - 1.08)	0.94 (0.80 - 1.12)
35 - <45	0.99 (0.90 - 1.09)	0.96 (0.83 - 1.10)	0.92 (0.76 - 1.10)
45+	0.81** (0.73 - 0.91)	1.04 (0.90 - 1.19)	1.07 (0.90 - 1.27)
Prior arrest (ref. no prior)			
1	0.82** (0.73 - 0.92)	1.02 (0.89 - 1.17)	1.02 (0.85 - 1.23)
2	0.79** (0.70 - 0.88)	0.83* (0.71 - 0.97)	0.84 (0.68 - 1.04)
3	0.68** (0.61 - 0.76)	0.78** (0.68 - 0.90)	0.77** (0.65 - 0.93)
Drug charge	1.88** (1.52 - 2.31)	1.06 (0.84 - 1.34)	1.27 (0.96 - 1.68)
Property charge	1.38** (1.23 - 1.55)	0.74** (0.61 - 0.90)	1.19 (0.98 - 1.45)
Violent charge	0.80** (0.70 - 0.91)	1.10 (0.94 - 1.29)	0.70** (0.56 - 0.88)
DUI charge	2.43** (1.94 - 3.03)	1.43** (1.21 - 1.69)	2.23** (1.77 - 2.82)
Jail sentence length (ref. 1–30 days)			
31 – 60	0.56** (0.48 - 0.65)	0.71** (0.60 - 0.83)	0.69** (0.58 - 0.82)
61 – 90	0.73** (0.64 - 0.83)	0.75** (0.60 - 0.92)	0.76* (0.59 - 0.98)
91+	0.32** (0.28 - 0.36)	0.70** (0.56 - 0.88)	0.38** (0.28 - 0.50)
Observations	20,666	11,398	6,384

Notes: ** p<0.01, * p<0.05. AI refers to American Indian. Race equals 0 if the recorded race was White, 1 if the recorded race is AI, and 2 for all other race/ethnicities (i.e., Asian, Black, other, unknown). The control variables for all models include gender, age, prior arrests, jail sentence length, charge type, and county unemployment, as well as county and month fixed effects. All standard errors are clustered by county and month-year. The periods for the binary indicator are pre-reform (July 1, 2011 – June 30, 2013) and post-reform (July 1, 2013 – June 30, 2015).

eTable 4F: Logistic regression models examining association between 6-month interval reform indicator, race, and fine assessment including all persons regardless of recorded race

Variables	Urban	Rural, No Indian Country	Rural, Part Indian Country
	OR (95% CI)	OR (95% CI)	OR (95% CI)
Reform (ref. pre-reform)			
6-months post	0.69 (0.45 - 1.06)	1.23 (0.88 - 1.72)	0.80 (0.50 - 1.29)
12-months post	0.20** (0.13 - 0.32)	0.12** (0.08 - 0.18)	0.15** (0.09 - 0.23)
18-months post	0.07** (0.04 - 0.12)	0.07** (0.05 - 0.10)	0.12** (0.08 - 0.19)
24-months post	0.10** (0.06 - 0.16)	0.07** (0.05 - 0.11)	0.11** (0.07 - 0.19)
Reform*race (ref. White)			
6-months post*AI	0.59* (0.39 - 0.89)	0.45** (0.29 - 0.68)	0.56* (0.35 - 0.88)
12-months post*AI	1.16 (0.84 - 1.60)	0.87 (0.62 - 1.21)	0.78 (0.53 - 1.16)
18-months post*AI	1.61** (1.17 - 2.23)	0.89 (0.59 - 1.34)	0.72 (0.49 - 1.07)
24-months post*AI	1.92** (1.38 - 2.68)	1.14 (0.81 - 1.62)	0.69 (0.47 - 1.01)
6-months post*Other	0.96 (0.71 - 1.31)	1.03 (0.57 - 1.87)	1.98 (0.46 - 8.49)
12-months post*Other	1.03 (0.71 - 1.48)	0.85 (0.54 - 1.34)	0.90 (0.38 - 2.15)
18-months post*Other	1.27 (0.86 - 1.87)	0.96 (0.66 - 1.42)	0.39** (0.20 - 0.75)
24-months post*Other	0.93 (0.70 - 1.24)	0.94 (0.66 - 1.34)	0.44* (0.22 - 0.91)
Unemployment rate	0.65* (0.44 - 0.96)	0.75* (0.58 - 0.97)	0.77* (0.61 - 0.98)
Male (ref. female)	0.96 (0.89 - 1.03)	1.02 (0.92 - 1.14)	1.22** (1.05 - 1.41)
Age (ref. 18 - <25 years)			
25 - <35	0.98 (0.90 - 1.07)	1.00 (0.88 - 1.14)	0.93 (0.78 - 1.11)
35 - <45	1.01 (0.92 - 1.11)	1.03 (0.88 - 1.19)	0.90 (0.75 - 1.09)
45+	0.82**(0.73 - 0.91)	1.09 (0.94 - 1.26)	1.05 (0.88 - 1.25)
Prior arrest (ref. no prior)			
1	0.81** (0.72 - 0.90)	1.01 (0.87 - 1.16)	1.03 (0.86 - 1.24)
2	0.78** (0.70 - 0.87)	0.79** (0.67 - 0.94)	0.81 (0.65 - 1.01)
3	0.67** (0.60 - 0.75)	0.76** (0.66 - 0.88)	0.79* (0.65 - 0.95)
Drug charge	1.94** (1.57 - 2.39)	1.09 (0.85 - 1.40)	1.35* (1.00 - 1.81)
Property charge	1.41** (1.26 - 1.59)	0.73** (0.59 - 0.91)	1.17 (0.95 - 1.44)
Violent charge	0.79** (0.70 - 0.90)	1.04 (0.88 - 1.23)	0.67** (0.52 - 0.84)
DUI charge	2.53** (2.02 - 3.17)	1.43** (1.19 - 1.71)	2.26** (1.77 - 2.89)
Jail sentence length (ref. 1–30 days)			
31 – 60	0.55** (0.48 - 0.63)	0.65** (0.55 - 0.77)	0.67** (0.56 - 0.80)
61 – 90	0.69** (0.61 - 0.79)	0.67** (0.54 - 0.83)	0.76* (0.59 - 0.99)
91+	0.30** (0.27 - 0.34)	0.64** (0.51 - 0.81)	0.37** (0.28 - 0.50)
Observations	20,666	11,398	6,384

Notes: ** p<0.01, * p<0.05. AI refers to American Indian. Race equals 0 if the recorded race was white, 1 if the recorded race is AI, and 2 for all other race/ethnicities (i.e., Asian, Black, other,

unknown). The control variables for all models include gender, age, prior arrests, jail sentence length, charge type, and county unemployment, as well as county and month fixed effects. All standard errors are clustered by county and month-year. The periods for the binary indicator are pre-reform (July 1, 2011 – June 30, 2013) and post-reform (July 1, 2013 – June 30, 2015).

eTable 4G: Linear probability models examining association between reform, race, and fine assessment using a three-way interaction term

	Model 1: Rural (Ref. Urban)	Model 2: Indian Country (Ref. No Indian Country)
	β (95% CI)	β (95% CI)
Panel A: Binary reform indicator		
County	0.18* (0.04 - 0.32)	-0.19** (-0.30 - -0.07)
Reform (ref. pre-reform)	-0.26** (-0.32 - -0.19)	-0.28** (-0.33 - -0.23)
AI (ref. White)	-0.16** (-0.19 - -0.13)	-0.10** (-0.14 - -0.06)
County * Reform	0.01 (-0.06 - 0.08)	0.07* (0.01 - 0.14)
County * AI	0.07** (0.03 - 0.12)	0.03 (-0.03 - 0.08)
Reform * AI	0.18** (0.13 - 0.24)	0.05 (-0.00 - 0.11)
County * Reform * AI	-0.16** (-0.22 - -0.09)	-0.09* (-0.17 - -0.01)
Panel B: 6-month interval reform indicator		
County	0.06 (-0.06 - 0.17)	-0.15** (-0.26 - -0.04)
Reform (ref. pre-reform)		
6-months post	-0.04 (-0.09 - 0.02)	0.02 (-0.02 - 0.06)
12-months post	-0.31** (-0.40 - -0.22)	-0.37** (-0.45 - -0.29)
18-months post	-0.50** (-0.57 - -0.43)	-0.46** (-0.53 - -0.40)
24-months post	-0.47** (-0.54 - -0.39)	-0.47** (-0.54 - -0.39)
American Indian (ref. White)	-0.17** (-0.20 - -0.14)	-0.10** (-0.14 - -0.07)
County * Reform		
6-months post	0.03 (-0.03 - 0.09)	-0.06* (-0.12 - -0.01)
12-months post	-0.02 (-0.12 - 0.08)	0.11* (0.01 - 0.20)
18-months post	0.05 (-0.03 - 0.14)	0.10* (0.02 - 0.18)
24-months post	0.05 (-0.04 - 0.14)	0.14** (0.04 - 0.23)
County * American Indian	0.08** (0.04 - 0.12)	0.03 (-0.02 - 0.08)
Reform * American Indian		
6-months post	0.04 (-0.05 - 0.14)	0.02 (-0.07 - 0.10)
12-months post	0.18** (0.10 - 0.25)	0.05 (-0.03 - 0.14)
18-months post	0.25** (0.18 - 0.32)	0.05 (-0.04 - 0.14)
24-months post	0.28** (0.21 - 0.36)	0.10* (0.01 - 0.18)
County * Reform * American Indian		
6-months post	-0.07 (-0.18 - 0.04)	-0.05 (-0.16 - 0.07)
12-months post	-0.14** (-0.25 - -0.04)	-0.10 (-0.22 - 0.02)
18-months post	-0.21** (-0.31 - -0.12)	-0.09 (-0.22 - 0.03)
24-months post	-0.24** (-0.33 - -0.14)	-0.15* (-0.27 - -0.03)
Observations	34,700	16,308

Notes: ** p<0.01, * p<0.05. Model 1 uses all counties in the dataset to examine rural vs. urban differences. The county indicator should be interpreted as the association between rural county (ref. urban) and fine assessment. The model does not consider if a county contains Indian Country. Model 2 examines these differences. We limit the analysis to rural counties because only one urban county contains Indian Country. The county indicator in this specification should

be interpreted as the association between Indian Country county (ref. No Indian Country county) and the likelihood of receiving a fine. AI refers to American Indian. AI equals 1 if the convicted person's race was recorded as American Indian and 0 if the recorded race was White. The control variables for all models include gender, age, prior arrests, jail sentence length, charge type, and county unemployment, as well as county and month fixed effects. All standard errors are clustered by county and month-year. The periods for the binary indicator are pre-reform (July 1, 2011 – June 30, 2013) and post-reform (July 1, 2013 – June 30, 2015). The periods for the 6-month interval indicator refer to the following dates: pre- reform: July 1, 2011 – June 30, 2013; 6-months post: July 1, 2013-December 31, 2013; 12-months post: January 1, 2014 – June 30, 2014; 18-months post: July 1, 2014 – December 31, 2014; 24-months post: January 1, 2015 – June 30, 2015.

Table 4H: Marginal effect estimates of American Indian (Ref. White) on fine assessment before and after reform using multi-level model

	Urban	Rural, No Indian Country	Rural, Part Indian Country
Panel A: Binary reform indicator			
Before	-0.16** (-0.18 - -0.14)	-0.09** (-0.13 - -0.06)	-0.07** (-0.11 - -0.04)
After	0.02* (0.00 - 0.04)	-0.04* (-0.07 - -0.00)	0.10** (-0.13 - -0.06)
Panel B: 6-month interval reform indicator			
Pre-reform	-0.17** (-0.19 - -0.15)	-0.09** (-0.13 - -0.06)	-0.07** (-0.10 - -0.03)
6-months post	-0.12** (-0.16 - -0.08)	-0.07* (-0.14 - -0.01)	-0.08* (-0.15 - -0.02)
12-months post	0.01 (-0.03 - 0.05)	-0.03 (-0.10 - 0.03)	-0.10** (-0.16 - -0.04)
18-months post	0.08** (0.05 - 0.12)	-0.04 (-0.11 - 0.02)	-0.10** (-0.16 - -0.03)
24-months post	0.12** (0.08 - 0.15)	0.00 (-0.06 - 0.06)	-0.12** (-0.18 - -0.06)
Observations	34,700		

Notes: ** p<0.01, * p<0.05. Marginal effects derived from multi-level linear probability model. The multi-level model was structured as follows: convictions (level 1) nested in counties (level 2) nested in county types (level 3). The level 1 control variables were gender, age, prior arrests, jail sentence length, charge type, and month of arrest. The level 2 control variable was the monthly unemployment rate. The periods for the binary indicator are pre-reform (July 1, 2011 – June 30, 2013) and post-reform (July 1, 2013 – June 30, 2015). The periods for the 6-month interval indicator refer to the following dates: pre- reform: July 1, 2011 – June 30, 2013; 6-months post: July 1, 2013-December 31, 2013; 12-months post: January 1, 2014 – June 30, 2014; 18-months post: July 1, 2014 – December 31, 2014; 24-months post: January 1, 2015 – June 30, 2015.

Table 4I: Logistic regression models examining association between binary reform indicator and fine assessment with multiple imputation

	Urban	Rural, No Indian Country	Rural, Part Indian Country
VARIABLES	OR (95% CI)	OR (95% CI)	OR (95% CI)
Reform (ref. pre-reform)	0.66 (0.42 - 1.04)	0.30** (0.23 - 0.39)	0.25** (0.19 - 0.34)
Unemployment rate	1.81* (1.13 - 2.92)	1.24 (0.96 - 1.62)	0.91 (0.71 - 1.16)
Male (ref. female)	0.98 (0.91 - 1.05)	1.10 (1.00 - 1.21)	1.24** (1.08 - 1.44)
Age (ref. 18 - <25 years)			
25 - <35	0.97 (0.89 - 1.07)	0.94 (0.83 - 1.07)	0.98 (0.82 - 1.16)
35 - <45	0.99 (0.89 - 1.10)	0.95 (0.82 - 1.10)	0.96 (0.79 - 1.18)
45+	0.84** (0.75 - 0.94)	1.04 (0.91 - 1.18)	1.15 (0.96 - 1.37)
Prior arrest (ref. no prior)			
1	0.73** (0.65 - 0.82)	0.93 (0.81 - 1.07)	1.00 (0.83 - 1.21)
2	0.69** (0.61 - 0.79)	0.77** (0.66 - 0.89)	0.84 (0.68 - 1.04)
3	0.57** (0.50 - 0.64)	0.69** (0.61 - 0.79)	0.70** (0.58 - 0.84)
Drug charge	1.67** (1.38 - 2.02)	1.08 (0.83 - 1.42)	1.32* (1.00 - 1.75)
Property charge	1.32** (1.17 - 1.49)	0.74** (0.61 - 0.89)	1.18 (0.97 - 1.43)
Violent charge	0.69** (0.60 - 0.80)	1.07 (0.91 - 1.26)	0.70** (0.56 - 0.87)
DUI charge	2.11** (1.73 - 2.56)	1.48**	2.22** (1.77 - 2.78)
Jail sentence length (ref. 1–30 days)		(1.26 - 1.75)	
31 – 60	0.62** (0.53 - 0.72)	0.72** (0.60 - 0.85)	0.67** (0.56 - 0.80)
61 – 90	0.82** (0.72 - 0.94)	0.75** (0.61 - 0.93)	0.75* (0.58 - 0.97)
91+	0.53** (0.46 - 0.61)	0.77* (0.63 - 0.95)	0.48** (0.35 - 0.66)
Observations	19,986	11,530	6,338

Notes: ** $p<0.01$, * $p<0.05$. Prior arrest, drug charge, property charge, violence charge, dui charge, and race were used through multiple imputation to predict the missing values in the dependent variable for the 3,154 conviction that meet all other eligibility criteria except missing jail penalty information. The control variables for all models include gender, age, prior arrests, jail sentence length, charge type, and county unemployment, as well as county and month fixed effects. All standard errors are clustered by county and month-year. The periods for the binary indicator are pre-reform (July 1, 2011 – June 30, 2013) and post-reform (July 1, 2013 – June 30, 2015).

Table 4J: Logistic regression models examining association between 6-month interval reform indicator and fine assessment with multiple imputation

	Urban	Rural, No Indian Country	Rural, Part Indian Country
VARIABLES	OR (95% CI)	OR (95% CI)	OR (95% CI)
Reform (ref. pre-reform)			
6-months post	0.81 (0.55 - 1.19)	1.16 (0.87 - 1.55)	0.77 (0.53 - 1.11)
12-months post	0.33** (0.22 - 0.51)	0.19** (0.14 - 0.27)	0.19** (0.12 - 0.28)
18-months post	0.15** (0.09 - 0.26)	0.12** (0.09 - 0.17)	0.14** (0.10 - 0.21)
24-months post	0.22** (0.13 - 0.39)	0.13** (0.09 - 0.18)	0.14** (0.09 - 0.21)
Unemployment rate	0.72 (0.47 - 1.10)	0.83 (0.66 - 1.04)	0.76* (0.60 - 0.96)
Male (ref. female)	0.98 (0.91 - 1.05)	1.11* (1.00 - 1.22)	1.27** (1.10 - 1.48)
Age (ref. 18 - <25 years)			
25 - <35	0.98 (0.89 - 1.07)	0.97 (0.85 - 1.10)	0.96 (0.80 - 1.15)
35 - <45	1.00 (0.90 - 1.12)	1.01 (0.86 - 1.18)	0.95 (0.77 - 1.17)
45+	0.84** (0.76 - 0.94)	1.07 (0.93 - 1.22)	1.13 (0.94 - 1.35)
Prior arrest (ref. no prior)			
1	0.73** (0.64 - 0.82)	0.90 (0.78 - 1.04)	1.02 (0.84 - 1.23)
2	0.69** (0.61 - 0.78)	0.73** (0.63 - 0.85)	0.81 (0.66 - 1.01)
3	0.56** (0.50 - 0.62)	0.68** (0.59 - 0.77)	0.70** (0.58 - 0.85)
Drug charge	1.73** (1.42 - 2.10)	1.10 (0.83 - 1.45)	1.39* (1.04 - 1.86)
Property charge	1.36** (1.21 - 1.54)	0.74** (0.61 - 0.90)	1.16 (0.95 - 1.42)
Violent charge	0.69** (0.60 - 0.79)	1.03 (0.87 - 1.22)	0.67** (0.53 - 0.84)
DUI charge	2.17** (1.78 - 2.64)	1.50** (1.25 - 1.78)	2.22** (1.76 - 2.80)
Jail sentence length (ref. 1–30 days)			
31 – 60	0.61** (0.52 - 0.70)	0.67** (0.57 - 0.80)	0.66** (0.55 - 0.79)
61 – 90	0.79** (0.69 - 0.90)	0.69** (0.55 - 0.86)	0.77 (0.60 - 1.00)
91+	0.52** (0.45 - 0.60)	0.74** (0.59 - 0.92)	0.49** (0.35 - 0.67)
Observations	19,986	11,530	6,338

Notes: ** p<0.01, * p<0.05. Prior arrest, drug charge, property charge, violence charge, dui charge, and race were used through multiple imputation to predict the missing values in the dependent variable for the 3,154 conviction that meet all other eligibility criteria except missing jail penalty information. The control variables for all models include gender, age, prior arrests, jail sentence length, charge type, and county unemployment, as well as county and month fixed effects. All standard errors are clustered by county and month-year. The periods for the 6-month interval indicator refer to the following dates: pre- reform: July 1, 2011 – June 30, 2013; 6-months post: July 1, 2013-December 31, 2013; 12-months post: January 1, 2014 – June 30, 2014; 18-months post: July 1, 2014 – December 31, 2014; 24-months post: January 1, 2015 – June 30, 2015.

Table 4K: Logistic regression models examining association between binary reform indicator, race, and fine assessment with multiple imputation for dependent variable

	(1)	(3)	(5)
VARIABLES	Binary	Binary	Binary
Reform (ref. pre-reform)	0.46** (0.29 - 0.74)	0.27** (0.21 - 0.36)	0.24** (0.17 - 0.34)
Reform*AI	1.20* (1.02 - 1.42)	0.81* (0.69 - 0.96)	0.68** (0.55 - 0.84)
Unemployment rate	1.84** (1.16 - 2.90)	1.25 (0.96 - 1.62)	0.90 (0.70 - 1.16)
Male (ref. female)	0.95 (0.88 - 1.03)	1.05 (0.96 - 1.16)	1.16* (1.00 - 1.34)
Age (ref. 18 - <25 years)			
25 - <35	0.96 (0.88 - 1.05)	0.94 (0.83 - 1.07)	0.96 (0.81 - 1.14)
35 - <45	0.99 (0.89 - 1.10)	0.93 (0.81 - 1.08)	0.92 (0.75 - 1.12)
45+	0.83** (0.75 - 0.93)	1.00 (0.88 - 1.14)	1.07 (0.90 - 1.28)
Prior arrest (ref. no prior)			
1	0.73** (0.65 - 0.83)	0.95 (0.83 - 1.09)	1.03 (0.85 - 1.25)
2	0.70** (0.62 - 0.80)	0.78** (0.67 - 0.91)	0.88 (0.71 - 1.09)
3	0.60** (0.53 - 0.67)	0.73** (0.65 - 0.84)	0.77** (0.64 - 0.93)
Drug charge	1.59** (1.31 - 1.93)	1.09 (0.83 - 1.42)	1.27 (0.96 - 1.68)
Property charge	1.27** (1.12 - 1.43)	0.74** (0.61 - 0.89)	1.20 (0.98 - 1.45)
Violent charge	0.65** (0.56 - 0.75)	1.11 (0.94 - 1.30)	0.70** (0.56 - 0.87)
DUI charge	1.96** (1.61 - 2.37)	1.46** (1.24 - 1.73)	2.11** (1.67 - 2.66)
Jail sentence length (ref. 1–30 days)			
31 – 60	0.61** (0.52 - 0.71)	0.72** (0.61 - 0.85)	0.68** (0.57 - 0.81)
61 – 90	0.80** (0.70 - 0.91)	0.75** (0.60 - 0.93)	0.76* (0.59 - 0.98)
91+	0.53** (0.46 - 0.61)	0.76** (0.62 - 0.94)	0.48** (0.35 - 0.65)
Observations	19,986	11,530	6,338

Notes: ** p<0.01, * p<0.05. Prior arrest, drug charge, property charge, violence charge, dui charge, and race were used through multiple imputation to predict the missing values in the dependent variable for the 3,154 conviction that meet all other eligibility criteria except missing jail penalty information. AI refers to American Indian. AI equals 1 if the convicted person's race was recorded as American Indian and 0 if the recorded race was White. The control variables for all models include gender, age, prior arrests, jail sentence length, charge type, and county unemployment, as well as county and month fixed effects. All standard errors are clustered by county and month-year. The periods for the binary indicator are pre-reform (July 1, 2011 – June 30, 2013) and post-reform (July 1, 2013 – June 30, 2015).

Table 4L: Logistic regression models examining association between 6-month interval reform indicator, race, and fine assessment with multiple imputation for dependent variable

	Urban	Rural, No Indian Country	Rural, Part Indian Country
VARIABLES	OR (95% CI)	OR (95% CI)	OR (95% CI)
Reform (ref. pre-reform)			
6-months post	0.75 (0.51 - 1.12)	1.17 (0.86 - 1.58)	0.80 (0.51 - 1.25)
12-months post	0.24** (0.15 - 0.37)	0.17** (0.12 - 0.24)	0.17** (0.11 - 0.26)
18-months post	0.09** (0.06 - 0.16)	0.11** (0.08 - 0.16)	0.13** (0.09 - 0.21)
24-months post	0.12** (0.07 - 0.21)	0.11** (0.08 - 0.16)	0.13** (0.08 - 0.21)
Reform*AI (ref. White)			
6-months post	0.61** (0.42 - 0.88)	0.52** (0.35 - 0.78)	0.55* (0.35 - 0.88)
12-months post	1.12 (0.80 - 1.55)	0.82 (0.59 - 1.13)	0.73 (0.50 - 1.06)
18-months post	1.64** (1.21 - 2.24)	0.78 (0.52 - 1.17)	0.66* (0.45 - 0.97)
24-months post	1.92** (1.37 - 2.68)	1.01 (0.72 - 1.41)	0.64*(0.44 - 0.94)
Unemployment rate	0.71 (0.48 - 1.04)	0.82 (0.66 - 1.02)	0.75* (0.59 - 0.96)
Male (ref. female)	0.95 (0.88 - 1.02)	1.06 (0.96 - 1.17)	1.19* (1.02 - 1.38)
Age (ref. 18 - <25 years)			
25 - <35	0.97 (0.88 - 1.06)	0.97 (0.85 - 1.10)	0.94 (0.79 - 1.12)
35 - <45	1.00 (0.90 - 1.12)	0.99 (0.85 - 1.16)	0.91 (0.74 - 1.12)
45+	0.83** (0.75 - 0.93)	1.03 (0.90 - 1.18)	1.05 (0.88 - 1.27)
Prior arrest (ref. no prior)			
1	0.72** (0.64 - 0.81)	0.92 (0.80 - 1.06)	1.05 (0.87 - 1.28)
2	0.69** (0.61 - 0.79)	0.75** (0.64 - 0.88)	0.86 (0.69 - 1.07)
3	0.58** (0.52 - 0.65)	0.72** (0.63 - 0.82)	0.78* (0.65 - 0.95)
Drug charge	1.65** (1.36 - 2.01)	1.10 (0.83 - 1.46)	1.34* (1.00 - 1.79)
Property charge	1.30** (1.15 - 1.46)	0.74** (0.61 - 0.91)	1.18 (0.96 - 1.45)
Violent charge	0.64** (0.55 - 0.74)	1.07 (0.90 - 1.26)	0.67** (0.53 - 0.85)
DUI charge	2.01** (1.66 - 2.44)	1.47** (1.23 - 1.76)	2.11** (1.66 - 2.67)
Jail sentence length (ref. 1–30 days)			
31 – 60	0.60** (0.52 - 0.69)	0.68** (0.57 - 0.80)	0.67** (0.56 - 0.81)
61 – 90	0.76** (0.67 - 0.87)	0.69** (0.55 - 0.85)	0.77 (0.60 - 1.00)
91+	0.51** (0.45 - 0.59)	0.73** (0.58 - 0.91)	0.48** (0.35 - 0.66)
Observations	19,986	11,530	6,338

Notes: ** p<0.01, * p<0.05. Prior arrest, drug charge, property charge, violence charge, dui charge, and race were used through multiple imputation to predict the missing values in the dependent variable for the 3,154 conviction that meet all other eligibility criteria except missing jail penalty information. AI refers to American Indian. AI equals 1 if the convicted person's race was recorded as American Indian and 0 if the recorded race was White. The control variables for all models include gender, age, prior arrests, jail sentence length, charge type, and county unemployment, as well as county and month fixed effects. All standard errors are clustered by county and month-year. The periods for the 6-month interval indicator refer to the following

dates: pre- reform: July 1, 2011 – June 30, 2013; 6-months post: July 1, 2013-December 31, 2013; 12-months post: January 1, 2014 – June 30, 2014; 18-months post: July 1, 2014 – December 31, 2014; 24-months post: January 1, 2015 – June 30, 2015.

eTable 5: Generalized linear model examining the association between the fine amount, race, and fine amount among misdemeanor convictions assessed a fine

	Urban	Rural, No Indian Country	Rural, Part Indian Country
	(95% CI)	(95% CI)	(95% CI)
Panel A: Binary reform indicator			
Reform (ref. pre-reform)	-0.05 (-0.09 - 0.00)	-0.02 (-0.05 - 0.00)	-0.02 (-0.07 - 0.03)
Panel B: 6-month interval reform indicator			
Reform (ref. pre-reform)			
6-months post	-0.07*(-0.12 -0.01)	-0.04* (-0.08 - -0.01)	-0.05 (-0.12 - 0.02)
12-months post	-0.02 (-0.08 - 0.04)	-0.00 (-0.05 - 0.05)	-0.01 (-0.09 - 0.06)
18-months post	-0.05 (-0.13 - 0.03)	0.02 (-0.03 - 0.08)	0.04 (-0.05 - 0.14)
24-months post	0.04 (-0.03 - 0.11)	-0.04 (-0.10 - 0.01)	-0.01 (-0.10 - 0.07)
Observations	9,862	7,135	4,229

Notes: ** $p<0.01$, * $p<0.05$. The control variables for all models include gender, age, prior arrests, jail sentence length, charge type, and county unemployment, as well as county and month fixed effects. All standard errors are clustered by county and month-year. The periods for the binary indicator are pre-reform (July 1, 2011 – June 30, 2013) and post-reform (July 1, 2013 – June 30, 2015). The periods for the 6-month interval indicator refer to the following dates: before reform: July 1, 2011 – June 30, 2013; 6-months post: July 1, 2013-December 31, 2013; 12-months post: January 1, 2014 – June 30, 2014; 18-months post: July 1, 2014 – December 31, 2014; 24-months post: January 1, 2015 – June 30, 2015.

References

18 USC § 1151, (1949). https://www.law.cornell.edu/uscode/text/18/1151

40 CFR § 171.3, (2017). https://www.law.cornell.edu/cfr/text/40/171.3

National Congress of American Indians (NCAI). (2019). *NCAI Response to Usage of the Term, "Indian Country" | NCAI*. https://www.ncai.org/news/articles/2019/12/27/ncai-response-to-usage-of-the-term-indian-country

State of South Dakota. (2021). *South Dakota GIS Data*. https://opendata2017-09-18t192802468z-sdbit.opendata.arcgis.com/

The United States Attorney's Office: District of South Dakota. (2022, March 24). *Indian Country*. https://www.justice.gov/usao-sd/indian-country#:~:text=They%20are%20Santee%20Dakota%20people,Indian%20Reorganization%20Act%20of%201934.

United States Department of Agriculture. (n.d.). *Rural-Urban Continuum Codes*. Retrieved May 5, 2022, from https://ers.usda.gov/data-products/rural-urban-continuum-codes/

U.S. Census Bureau, Department of Commerce. (2021). *TIGER/Line Shapefile, 2017, nation, U.S., Current American Indian Tribal Subdivision (AITS) National*. https://catalog.data.gov/dataset/tiger-line-shapefile-2017-nation-u-s-current-american-indian-tribal-subdivision-aits-national

U.S. Department of the Interior: Indian Affairs. (n.d.). *Bureau of Indian Affairs Land Area Representation Dataset*. https://www.bia.gov/bia/ots/dpmc/bogs
